# Extraction, Phytochemical profile, and neuroprotective activity of *Phyllanthus emblica* fruit extract against sodium valproate-induced postnatal autism in BALB/c mice

**DOI:** 10.1016/j.heliyon.2024.e34992

**Published:** 2024-07-20

**Authors:** Balaji Gouda, Sukesh Narayan Sinha, Rajendra Sangaraju, Tien Huynh, Shashikala Patangay, Surekha Venkata Mullapudi, Sathish Kumar Mungamuri, Pradeep B. Patil, Madhusudhana Chary Periketi

**Affiliations:** a Division of Food Safety, Indian Council of Medical Research-National Institute of Nutrition, Jamai-Osmania, Tarnaka, Hyderabad, Telangana- 500007, India; b Head of Biology, Department of Biosciences and Food Technology, STEM College, RMIT University, PO Box 71, Bundoora, VIC 3083, Australia; c Department of Pharmacy, University College of Technology, Osmania University, Hyderabad-500027, India; d Division of Pathology and Microbiology, Indian Council of Medical Research-National Institute of Nutrition, Jamai-Osmania, Tarnaka, Hyderabad, Telangana- 500007, India; e Animal Facility Division, Indian Council of Medical Research-National Institute of Nutrition, Jamai-Osmania, Tarnaka, Hyderabad, Telangana- 500007, India; f SEM Facility, Cell Biology Division, Indian Council of Medical Research-National Institute of Nutrition, Jamai-Osmania, Tarnaka, Hyderabad, Telangana- 500007, India

**Keywords:** Autism spectrum disorder, Valproic acid, Ethyl acetate fraction of amla, Oxidative stress, Serotonergic and dopaminergic receptor proteins, Neuroprotective activity

## Abstract

The aim of the present study was to evaluate the effect of the ethyl acetate fraction of amla (EAFA) extract on valproic acid (VPA)-induced postnatal autism in BALB/c mice. Our study revealed that mice treated with VPA on postnatal day 14 (PND14) showed significant abnormal behaviours such as social interaction, social affiliation, anxiety, and motor coordination compared to the control group, while EAFA extract treatment (100 mg/kg) ameliorated these symptoms. Our study highlights the protective effect of EAFA extract on improving behavioural alterations, significantly restoring anti-oxidative enzymes such as GST and GR, and reducing MDA and NO levels. Furthermore, the EAFA-treated group significantly lowered the proinflammatory markers (IL-1β and TNF-α) and the expression of up-regulated 5-HT1D, 5-HT2A, and D2 receptor proteins. Based on histopathological studies, the percentage of neuronal injury in the EAFA-treated group as well as cellular structural changes were reduced using SEM analysis. In conclusion, the present study suggests that treatment with EAFA extract ameliorates VPA-induced autism due to its anti-oxidant and neuroprotective activity.

## 1 Introduction

Autism spectrum disorders (ASD) are a group of neurodevelopmental disorders that are characterised by difficulties in speech, repetitive behaviour, and social interaction. Patients with ASD display cognitive dysfunction, which manifests as deficits in spatial working memory and facial or object identification [1]. ASD is associated with several co-morbid features, including anxiety, seizure activity, motor abnormalities, aggressive behaviour, and sleep difficulties. According to earlier research, ASD affects 2–3 times more males than females [2]. ASD in children may be more likely in some cases due to a combination of risk factors, including genetics, prenatal viral infections, and medication therapies like valproic acid [3].

Valproic acid (VPA) is primarily used to treat several neurological conditions, such as epilepsy, resistant depression, and migraine prophylaxis [4], and is well known to cause foetal-valproate syndrome [5]. Early postnatal days of brain development in mice and rats are comparable to the stages of foetal brain development in humans during the third trimester of pregnancy [6]. PND (Post Natal Day) 14 is a critical time when the cerebellum, striatum, and hippocampus are considered to undergo neuronal migration, differentiation, myelination, synaptogenesis, and gliogenesis [7]. VPA is exposed to prenatal and postnatal rodents that cause autism-like neurobehavioral abnormalities [8]. It has been documented that giving VPA to rodents on PND 14 results in intrusions and neurodevelopmental regressions, which in turn produce behavioural retardations. Additionally, VPA treatment in rodents on PND14 causes cell death in the cerebellum and hippocampus [5].

In the initial stages of neurodevelopment, the neurotransmitter serotonin (5-hydroxytryptamine, 5-HT) is extremely important. In particular, 5-HT has been shown to control a number of neurobiological processes, such as synaptic transmission, synaptic plasticity, neurite outgrowth, and dendritic spine shape [9]. In autistic patients, repetitive behaviours may be related to hypersensitivity of the 5-HT1D receptors [10]. Moreover, 5-HT2A receptors play an essential role in central nervous system (CNS) illnesses [11]. Apart from this, dopamine 2 receptors (D2R) may be linked to various symptoms of ASD, including stereotypy, other repetitive behaviours, and language difficulty [12]. Also, earlier reports demonstrated that the D2 receptor expression was raised in the animal model of VPA [13].

Oxidative stress is one of the important biomarkers in both human and animal post-mortem brains with ASD [14]. There is additional evidence of aberrant cytokine release in the peripheral blood of ASD patients [15]. The cytokine profiles of IL-1β and TNF-α have been found to be elevated in the population with ASD, according to numerous studies [16].

*Phyllanthus emblica Linn. or Emblica officinalis* Gaertn. (Euphorbeaceae), also known as Indian gooseberry or amla, is distributed in tropical and subtropical areas of India, China, Indonesia, and the Malay Peninsula. It is a rich dietary source of vitamin C (l-ascorbic acid), minerals, amino acids, vitamins, fixed oils, and tannins, and also contains higher amounts of polyphenols like ethyl gallate, gallic acid, methyl gallate, ellagic acid, and flavonoids like rutin and quercetin [17]. According to pharmacological research studies, amla possesses analgesic, anti-tussive, anti-atherogenic, adaptogenic, cardiac, gastro-protective, nephroprotective, neuro-protective, anti-cancer, anti-inflammatory, free radical scavenging [18], chemo-preventive, and immunomodulatory activities [19].

ASD treatment begins in early childhood and continues throughout life. To date, no medication has been proven to be effective in treating the core symptoms of autism spectrum disorder (ASD); only symptom-based treatment is available. Given that the FDA-approved medications aripiprazole and risperidone act on D2 receptors and ameliorate stereotypies in ASD patients, this hypothesis may already be confirmed [12]. As of now, there are no FDA-approved drugs specifically for treating ASD. However, the extended use of currently available medications for autism treatment has been linked to harmful outcomes. It is crucial to discover phytoconstituents that possess greater therapeutic potential and fewer side effects than the currently marketed drugs to meet unmet needs. Therefore, the primary objective of the present therapy should be to develop a medication with a potent safety, economic feasibility, and tolerability profile. Herbal medications are especially suitable for treating chronic illnesses or disorders since they have been shown to have little to no adverse effects in autism treatment. Based on this assumption, we have chosen a phytomedicine-like amla fruit for the treatment of autism-like symptoms.

BALB/c mice are less responsive to social contact in VPA-induced autism than several other inbred mouse strains, including C57BL/6J mice [5,11]. According to our previous reports, postnatal BALB/c mice were exposed to VPA, and it was observed that male animals were more significantly affected than females [5,11].

This study aims to evaluate the ameliorating effect of the ethyl acetate fraction of amla (EAFA) on VPA-induced oxidative stress, behavioural, histopathological, pro-inflammatory, serotonin (5-HT1D and 5-HT2A), and dopamine 2 receptor protein (D2) alterations in BALB/c mice.

## 2 Materials and methods

### 2.1 Chemicals and materials

The procurement of the chemicals for the study involved the purchase of sodium valproate (with a purity level of at least 94.0 %) from Sigma-Aldrich, USA, while Aculife Company supplied the normal saline and water for injection (WFI). Additionally, formalin and N-(1-naphthyl)-ethylenediamine dichloride (NED) were purchased from SD Fine Chemicals, Mumbai, India, and all organic solvents were acquired from SRL Chemicals. For the analysis, J.T. Bakers provided all the solvents, which were of LC-MS grade. All standards (quercetin (purity ≥95.0 %), rutin (purity ≥94.0 %), calcium pantothenate (purity ≥98.0 %), gallic acid (purity ≥97.0 %), l-ascorbic acid (purity ≥99.0 %), reduced-glutathione (GSH), glutathione-S- transferase (GST), 1-chloro-2,4-dinitrobenzene (CDNB), 2-thiobarbituric acid (TBA), Griess reagent, malondialdehyde (MDA), 5,5′dithio-bis (2-nitro benzoic acid) (DNTB), trichloroacetic acid (TCA), β-nicotinamide adenine dinucleotide hydrate (NADH), *o*-dianisidine dihydrochloride and other reagents were obtained from Sigma Aldrich. ProLiant New Zealand Ltd. provided the bovine serum albumin (BSA), while Lobachemie Pvt. Ltd., Mumbai, supplied the sulphanilamide. The BCA protein assay kit was obtained from GCC Biotech, and the ELISA assay kits such as TNF-α and IL-1β were bought from Krishgen Biosystems, Mumbai, India.

### 2.2 Plant material

The dried fruits of *Phyllanthus emblica* (Euphorbiaceae family) were procured locally in Hyderabad, India, and authenticated by Prof. H. Ramakrishna of the Department of Botany, Osmania University, Hyderabad, and Telangana, India. A voucher specimen (no. 322) from the Botany Department of S.V. University, Tirupati, Andhra Pradesh, India. The fruits were washed with tap water and rinsed with distilled water to remove potential contaminants. Then, clean fruits were chopped and dried in an oven at 60 °C. We ground dried fruits into smaller parts for extraction [20].

### 2.3 Preparation of dried fruit extract of Phyllanthus emblica

The extraction method for *Phyllanthus emblica* is the same as previously described [18]. Briefly, using an electronic grinder, the dried fruits were ground into a fine powder. Then, 100 g of amla was extracted with 70 % of 500 mL of methanol at room temperature while being continuously stirred on a magnetic stirrer for an hour. The Whatman filter paper was used to filter the raw extract, and then a rota evaporator was used to concentrate it. A 30 g dry extract was redissolved in 750 mL of HPLC-grade water, then treated with an equal volume of hexane resultants to defat the extract, and then treated with an equal volume of chloroform resultants to remove highly nonpolar compounds. Finally, ethyl acetate was used to extract the aqueous layer. Lastly, the ethyl acetate fraction layer was evaporated under reduced pressure on a rotary evaporator to get the ethyl acetate fraction of amla (EAFA). The percentage yield of EAFA extract is 6.02 % in 100 g of amla fruit dried powder. Based on previously reported acute toxicity studies on amla extracts, the safest dose was up to 5000 mg/kg BW [21]. Based on the prior investigation, the dose of the ethyl acetate fraction of amla extract (EAFA) at 100 mg/kg BW was chosen for the treatment group in the current study [22].

### 2.4 Ferric reducing antioxidant power assay (FRAP)

With a few modifications, the reducing power of the extracts was assessed using the technique outlined by Wang et al. (2019) [23]. Extract samples and l-ascorbic acid were dissolved in distilled water at a suitable concentration. Briefly, six different concentrations of 70 % methanol and EAFA extracts (0.25, 0.125, 0.0625, 0.03125, 0.015625, and 0.0078125 mg/mL) and l-ascorbic acid at the same concentrations were mixed with 1 mL of phosphate buffer (0.2 M, pH 6.6) and 1 mL of 1 % potassium ferricyanide. For 20 min, the mixture was incubated at 50 °C. The mixture was then centrifuged at 1000 rpm for 10 min after 1 mL of 10 % trichloroacetic acid (TCA) was added. The supernatant was collected. Then, 25 μL of ferric chloride (0.1 %) was added to the 75 μL of supernatant. Following a 10-min reaction, the solution's absorbance at OD700 nm was measured using a multimode microplate reader (Synergy H1 hybrid reader, Biotek). A better reduction activity is indicated by a higher OD absorbance value at 700 nm.

### 2.5 Nitric oxide (NO) scavenging assay

The nitric oxide scavenging ability of various extracts of amla was determined in triplicate by the Griess method.Briefly, sodium nitroprusside (100 μL of 10 mM) was added to 30 μL of EAFA and 70 % methanol amla extract (1 mg/mL in DMSO) and incubated at room temperature for 1 h. Then 50 μL of sulphanilamide (1 % in 5 % orthophosphoric acid) was added and incubated at room temperature for 10 min, followed by the addition of 50 μL of 0.1 % naphthyl ethylenediamine (NED). The contents were shaken well before measuring absorbance at 540 nm. The IC50 values calculated denote the concentration of sample required to decrease absorbance at 540 nm by 50 %. The IC50 value units were represented as mg/mL. l-ascorbic acid was used as a standard control.

### 2.6 Qualitative and quantitative analysis of EAFA extract

#### 2.6.1 Phytochemical profiling of EAFA extract using UPLC-ESI-Q-TOF-MS^E^

The identification of the different components in the EAFA was done by UPLC-ESI-Q-TOF-MSE analysis using an ACQUITY CSH-C18 column (100 mm × 2.1 mm, 1.8 μm particle size, Waters, Milford, MA, USA). The chromatographic separation was performed at 40 °C of column temperature and 8 °C of auto sampler temperature using the following method: The injection volume was 3 μL, the flow rate was 0.4 mL/min, and the ideal mobile phases were 0.1 % formic acid in water (solvent A) and 0.1 % formic acid in acetonitrile (solvent B). Here, a gradient solvent system was used: 0–1.0 min, 90 % A; 1.0–3.0 min, 90%–75 % A; 3.0–6.5 min, 75%–50 % A; 10.0–14.0 min, 50%–35 % A; 14.0–17.0 min, 35-10 % A; 17.0–19.0 min, 10-0% A; 0.0–23.0 min equilibration performed. The desolvation gas (high-purity nitrogen) was set at 850 L/h at 400 °C; at 50 L/h, the cone gas was set; and the temperature of the source was fixed at 150 °C for simultaneous MS and MS/MS analysis [24].

#### 2.6.2 UPLC-MS/MS quantification of phytochemical constituents in EAFA extract

To quantify the different components are present in the EAFA, LC-MS/MS analysis was performed by the standard method using the Xevo TQ-S micro-MS system coupled with Acquity H-class ultra-performance liquid chromatography (UPLC) (Waters, Milford, MA., USA). The Waters Acquity UPLC® analysis of the EAFA was performed using a CSH C18 column (100 mm × 2.1 mm, 1.7 μm particle size). The mobile phase used for the separation was made up of A (0.1 % formic acid in water) and B (0.1 % formic acid in acetonitrile), and the column temperature was fixed at 40 °C. The rate of gradient flow was maintained in the range of 0.3–0.400 mL/min, and the gradient solvent system was as follows: 0–2.5 min, 98 % A at flow 0.3 mL/min; 2.5–3 min, 70 % A at flow 0.300 mL/min; 3–6 min, 50 % A at flow 0.300 mL/min; 6–7.5 min, 35 % A at flow 0.30 mL/min; 7.5–8 min, 2 % A at flow 0.40 mL/min; 8–13.5 min, 2 % A at flow 0.40 mL/min; 13.5–17.5 min, 98 % A at flow 0.40 mL/min with a total run time of 23 min for multi-class analysis. The injection volume was 3 μL.

A pump, a detector, and a degasser are included with the UPLC. The Masslynx software was used to acquire the instruments and process the data (version 4.1). The mass was used in the automatic polarity switching mode with a positive and negative electrospray ionisation (ESI) source. A 150 °C source temperature was maintained. 3.00 kV was chosen as the capillary voltage; at 850 L/h, the de-solvation gas flow was set; and at 400 °C, the de-solvation temperature was held constant. Every analyte could be found, and the mass abundance ratio could be used to find the precursors and product ions. The multiple reaction monitoring (MRM) mode was utilised for scanning and obtaining large amounts of data for quantification, and it was also used to optimise the settings for all the analytes.

### 2.7 Animals

We kept BALB/c mice, both male and female, in plastic cages with open access to food and water ad libitum. We observed the emergence of the vaginal plug, regarded as embryonic day (ED) 0, daily in female mice. Postnatal day (PND) 0 was assigned to the day of birth. All experimental procedures were completed in compliance with the rules established by the committee for the purpose of control and supervision of experiments on animals (CPCSEA Reg. No. 154/G0/RBiBt-S/R-L/1999/CPCSEA). The temperature was maintained at 25 °C and the humidity at 55 %, while the lights were set to a 12-h light/12-h dark cycle. The Institutional Animal Ethics Committee looked over and approved this study. The National Institute of Nutrition in Hyderabad, India, conducted the study under the ethical number ICMR-NIN/IAEC/02/007/2019.

### 2.8 Experimental design

Nine distinct litters of animals were chosen and randomly divided into three groups; each group had six male animals (n = 6). VPA was dissolved in normal saline. A dose was calculated based on the animals' body weights (BW). On PND 14, sodium valproate (400 mg/kg BW) was administered subcutaneously to all animals except the control group [5,8,11,25].

Group I (Control): vehicle control group (only normal saline single dose at PND 14th day) + only (WFI) was administered orally and once daily for 27 days from PND 13th day to PND 40th day.

Group II (VPA): Disease control (VPA 400 mg/kg BW in normal saline, single dose at only PND 14th day, subcutaneous route) + only WFI was administered oral route, once daily for 27 days from PND 13th day to PND 40th day.

Group III (VPA + EAFA): Test control (VPA 400 mg/kg BW in normal saline, single dose at only PND 14th day, sub-cutaneous route) + EAFA 100 mg/kg BW dissolved in WFI was administered oral route, once daily for 27 days from PND 13th day to PND 40th day.

**Fig. 1:**
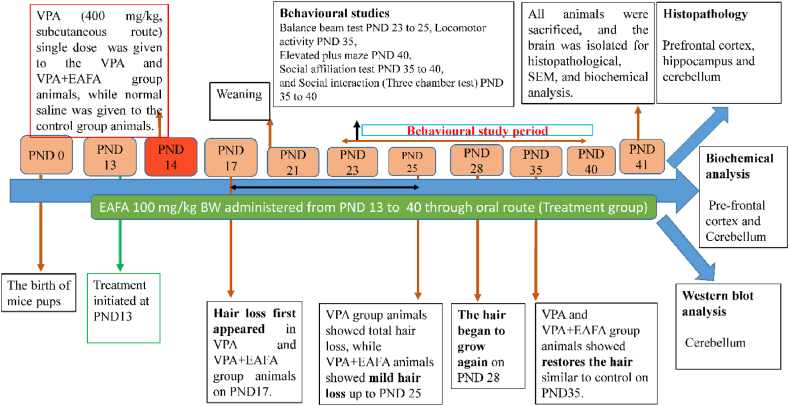
A detailed experimental design of the study, including VPA injection, behavioural tests, and sample collection for histopathological, immunohistochemical, antioxidant, and Western blot analysis.

During the experimentation period, all animal body weights were taken. Mice pups were subjected to behavioural testing to evaluate their motor coordination, locomotion, anxiety, social affiliation, and social interaction on various postnatal days up to postnatal day 40. All behavioural experiments were done between 9:00 and 15:00 (Fig. 1). On PND 41 Day, all experimental animals were sacrificed by cervical dislocation; brain tissue was washed with PBS and stored at 10 % buffered formalin for histopathological and 4 % formalin for IHC analysis; and tissues were kept in liquid nitrogen and stored at −80 °C for further biochemical, ELISA and Western blot analysis.

### 2.9 Behavioural models

#### 2.9.1 Balance beam test

A balancing beam test was conducted as previously reported to evaluate impairments of motor coordination [8,11]. Mice were positioned mid-way down an elevated beam that was 1 cm broad and 80 cm long. The room's lights were turned out, and a 60-W bulb lit up one side of the beam while the other led to an enclosed dark box (15 cm^3^) that was lined with bedding from the animal's home cage. On three consecutive days (from PND 23 to PND 25), every mouse has crossed a beam. The first two days were training days (PND 23 and PND 24), and the third day was the test day (PND 25). On the test day (PND 25), we videotaped the animals crossing the beam from the lit side to the dark box during each 60-s trial. From this video, we noted the number of foot slips, the distance travelled (centimetres (cm)), and the time (seconds).

#### 2.9.2 Actophotometer for locomotor activity

On PND 35, the locomotive activity test was performed on each mouse separately by using a digital actophotometer (Opto-Varimex-Minor, Columbus Instruments, Columbus, OH, USA). Three infrared emitters are placed on each x- and y-axis of this instrument, while an equal number of receivers are placed on the opposite side. Each mouse was placed inside the actophotometer for 5 min. The locomotor activity was defined as the number of beam breaks by the mouse [5].

#### 2.9.3 Elevated plus maze test

The elevated plus maze instrument was composed of wood and was used for evaluating anti-anxiety in the mice. It consisted of two open arms (25 cm × 5 cm) and two closed arms of the same dimensions, with opaque walls of 15 cm. The whole instrument was in an elevated position, i.e., 55 cm above ground level. On the 40th postnatal day, in the first instance, each mouse was allowed a period of 5 min to adjust to the environmental conditions. Immediately after the pre-test, each mouse was positioned on the central square of the maze, 5 cm × 5 cm, facing one of the open arms, and each mouse movement was monitored by videotape, recording its entries into the open arms and closed arms, as well as the time spent in the open and closed arms within a stipulated time period of 5 min [11].

The following data variables were measured by the following formula:

$$\%\;\text{time}\;\text{spent}\;\text{in}\;\text{open}\;\text{arms}=\frac{\text{Time}\;\text{spent}\;\text{in}\;\text{the}\;\text{open}\;\text{arm}\;\text{time}}{\;\text{Time}\;\text{spent}\;\text{in}\;\text{the}\;\text{open}\;\text{arm}+\text{time}\;\text{spent}\;\text{in}\;\text{the}\;\text{close}\;\text{arm}\;}\times 100$$

$$\%\;\text{entries}\;\text{in}\;\text{the}\;\text{open}\;\text{arms}=\frac{\text{Entries}\;\text{in}\;\text{the}\;\text{open}\;\text{arm}}{\;\text{Entries}\;\text{in}\;\text{the}\;\text{open}\;\text{arm}+\text{Entries}\;\text{in}\;\text{the}\;\text{close}\;\text{arm}\;}\times 100$$

An entry was defined as entering into one of the arms with all four paws. A decrease in percentage time spent and percentage (%) entries in open arms were considered as anxiety behaviours.

#### 2.9.4 Social affiliation

On PND 35–40, adolescent cage-mate affiliative behaviour was measured. The night before the experiment, the mice were put in separate cages so that they would interact more with each other during the experiment. Mice were matched based on their weight and gender. The subjects were placed for 15 min with their cage partner in a novel cage (42 x 42 × 21 cm), during which the mean duration of affiliative behaviours (side-to-side contact), social interaction (anogenital sniffing, nose-to-nose sniffing, and social grooming), and non-social cage exploration were analysed by a trained observer [26].

#### 2.9.5 Assessment of social interaction

A three-chamber social interaction testing apparatus (57 cm × 36 cm x 30 cm), as previously reported, was used to examine social interaction [14]. Briefly, the testing procedure involved three sessions. The initial 5-min session was the habituation phase for the treated mice. During the second 10-min session of the sociability phase, a stranger mouse was put into the chamber that was labelled “stranger,” while the other chamber was labelled “empty.” The final 10-min session was a social preference phase. During this phase, a novel mouse was put in the earlier-mentioned empty chamber and called the “novel chamber.” In this phase, the stranger chamber was referred to as the familiar chamber. In every session, the experiment mouse was free to explore the rooms. The time spent in both side chambers by the experiment mouse was noticed. Sociability and social preference were described as the sociability index (SI) and social preference index (SPI).

$$\text{SI}=\frac{\text{Time}\;\text{spent}\;\text{in}\;\text{stranger}\;\text{chamber}\;}{\text{Time}\;\text{spent}\;\text{in}\;\text{empty}\;\text{chamber}\;}$$

$$\text{SPI}=\frac{\text{Time}\;\text{spent}\;\text{in}\;\text{novel}\;\text{chamber}\;}{\text{Time}\;\text{spent}\;\text{in}\;\text{familiar}\;\text{chamber}\;}$$

### 2.10 Histopathology

All animals were sacrificed by cervical dislocation, and the brains were immediately removed and carefully washed with PBS to remove any mucous and debris. They were preserved in a buffered formalin solution of 10 % until the histological analysis. Then, the formalin-fixed tissues were processed for dehydration. After being run through various series of alcohol concentrations of 30, 50, 70, 80, 90, and 95 %, the tissues were cleansed in methyl benzoate before being embedded in paraffin wax. A rotary microtome (Leica Biosystems) was used to cut sections that were 3 μm thick. Harris hematoxylin (Anatech Ltd., Thermo Fisher Scientific, and Battle Creek, MI, USA) was used to stain the sections, and eosin diluted in 95 % alcohol was used as a counterstain. After dehydration and cleaning, the processed sections were mounted with DPx mountant. Quantification analysis of neuronal injury score (NIS) in the PFC (prefrontal cortex) by counting at least 900 cells per slide, subdivided into 5 fields, followed by neuronal injury in the hippocampus dentate gyrus (DG) by counting at least 500 cells per slide, subdivided into 3 fields, and neuronal injury in the hippocampus cornu ammonis (CA4) by counting at least 900 cells per slide, subdivided into 3 fields, and followed by neuronal injury score in the cerebellum of the Purkinje layer by counting at least 250 cells, subdivided into 15 fields chosen randomly. The scoring of H&E stained neurocytes was done under light microscopy at 40× magnification. At 40× magnification, photomicrographs of the section preparations were taken with a Cilika inverted microscope [27,28].

$$\text{NIS}\;\%=\frac{\text{Number}\;\text{of}\;\text{damaged}\;\text{cells}}{\;\text{Total}\;\text{number}\;\text{of}\;\text{calculated}\;\text{cells}\;}\times 100$$

### 2.11 Scanning electron microscope (SEM) analysis

The cellular morphology of the prefrontal cortex, hippocampus, and cerebellum of all groups was analysed by SEM according to the method of Gouda et al., 2022 (Gouda et al., 2022). The change in morphology was captured on camera using an SEM (S3400 N Hitachi Japan) at 15 KV with various magnifications.

### 2.12 Immunohistochemical studies

On the last day of the experiment, the dissected brains were fixed in 4 % formalin. The brains were then cleaned with absolute ethanol (3 times in 3 h), absolute ethanol with xylene (1:1) (twice in 1 h), and xylene (3 times in 20 min). Then, after 3 h of tissue saturation, the samples were embedded in paraffin blocks using liquid paraffin. 3 μm serial sections were cut using a microtome (Leica, Bensheim, Germany) and mounted on polysine histology slides (ThermoFisher Scientific, USA, 03801). The brain sections were deparaffinized in xylene and rehydrated in various concentrations of ethanol before immunohistochemistry (IHC) analysis. For epitope retrieval, the sections were boiled twice in citrate buffer (pH 6.0) in a microwave oven for 5 min. After cooling and washing with PBS, the endogenous peroxidase was blocked with a 3 % solution of H2O2 in distilled water, and the slides were incubated with horse serum to block the non-specific targets for 1 h before further adding the primary antibodies overnight at 4 °C. List of preparations for the primary antibodies: polyclonal anti-5-HT2A (catalogue no. MBS175200, 1:500) and polyclonal anti-5-HT1D (catalogue no. MBS241844, 1:100). The primary antibody and secondary antibodies were diluted in the 3 % normal horse serum. The primary antibodies were obtained from MyBioSource, Inc., San Diego, CA, USA. The avidin/biotin/peroxidase complex amplification system (VECTASTAIN® Elite® ABC Universal Kit Peroxidase, Horse Anti-Mouse/Rabbit IgG, catalogue no. PK-6200, Burlingame, CA, USA) with the Metal-Enhanced DAB Substrate Kit from Thermo Fisher Scientific, Battle Creek, MI, USA, was used to create immunostaining on the second day and to visualise the antigen-antibody combination. PBS was used to clean the sections. Hematoxylin was used as a counterstain after being washed with distilled water. The primary antibody was not used in the negative control section, which was carried out similarly. At 40× magnification, photomicrographs of the section preparations were taken with a microscope (Cilika Inverted Microscope). Microscopically, positive staining was defined by identifying brown pigmentation visually with the DAB substrate. The IHC examination was carried out by a microscopic investigator who had no prior knowledge of the experimental groups. ImageJ software (NIH, Bethesda, MD, USA) was used to measure the percentage relative protein expression of target proteins on the stained microscope slides based on the optical density. The densitometry analysis of each image was performed using Image J software for estimating the relative protein expression of target proteins such as 5-HDT1D and 5-HT2A in percentage based on five field areas of microscopic examination on the cerebral cortex and the hippocampus and ten field areas of microscopic examination on the cerebellum that were randomly selected from each section slide and automatically quantified for statistical analysis (Gouda et al., 2022).

### 2.13 Biochemical assessments in prefrontal cortex and cerebellum tissues

The prefrontal cortex and cerebellum parts of brain tissue homogenate were prepared as described by Sangaraju et al. (2021) [29]. and the supernatant obtained was used for the estimation of various antioxidant markers, like glutathione reductase (GR), glutathione-S-transferase (GST), and nitrate levels. The pellet was then resuspended in 10 % trichloroacetic acid and centrifuged at 1800×*g* for 10 min. The concentration of thiobarbituric acid reactive substance (TBARS) was estimated using the supernatant. Using BSA as a standard, a bicinchonic acid (BCA) assay method was used to estimate the total protein content in the homogenates of brain tissue. A colorimetric-based multimode microplate reader (Synergy H1 hybrid reader, Biotek) was used for biochemical assays.

#### 2.13.1 Assay for glutathione-S-transferase (GST)

The Sangaraju et al. (2021) method was used to estimate the GST activity [29]. Briefly, equal volumes of GSH and potassium phosphate buffer were combined with 20 μL of the tissue supernatant (0.1 M, PH 6.5). 10 μL of CDNB were added to this mixture, and the kinetic programme in the microplate reader was used to measure the rate of change in CDNB absorbance with time at 340 nm for 5 min at a 1 min interval. The activity was expressed as nmol of CDNB conjugated/min/mL.

#### 2.13.2 Glutathione reductase (GR)

The Sangaraju et al. (2021) method was used to measure GR activity. By measuring GR at 340 nm and recording the results as U/mg of protein, the enzyme activity was determined at a temperature of 25 °C [29].

#### 2.13.3 Estimation of nitrites and lipid peroxidation of brain tissues

Production of NO was determined by quantifying the level of nitrite (an indicator of NO) in the brain tissue supernatants using the Griess reagent, as followed by Sangaraju et al. (2019) [30]. Briefly, 100 μL of brain tissue supernatants were combined with 100 μL of Griess reagent, which contains 0.1 % sulphanilamide, 1 % naphthalenediamine dihydrochloride, and 2.5 % H3PO4. Followed by a 10-min incubation period at room temperature, and a microplate reader was used to measure absorbance at 540 nm. Using a sodium nitrite (NaNO2) standard curve represented as μM/g of tissue, the amount of nitrite in the sample was calculated.

Brain lipid peroxidation was assessed using the Sangaraju et al. (2021) method with slight modifications [29]. A total of 3 mL of the reaction mixture, which contained 1 mL of 10 % tissue homogenate, 1 mL of TCA (10 %), and 1 mL of TBA (0.87 %), was incubated in boiling water for 45 min. After cooling, the tubes were centrifuged at 2500×*g* for 10 min, and absorbance of the supernatant was measured at 532 nm. The rate of lipid peroxidation was determined by measuring MDA content and expressed as nmol MDA eq/g of tissue at 37 °C using a molar extinction coefficient of 1.56 x 105 M − 1 cm.

### 2.14 Estimation of pro-inflammatory cytokines

Each animal's prefrontal cortex and cerebellum of brain tissue were weighed, chopped, and then homogenised at a 10 % concentration in a solution of ice-cold phosphate buffered saline (PBS, pH 7.4) with a 1 % halt protease inhibitor cocktail. The tissue homogenate was centrifuged at 5000×*g* for 20 min at 4 °C, and the supernatant was used to estimate TNF-α and IL-1β using the ELISA kits (Krishgen Biosystems, Mumbai, India). The BCA protein assay kit (catalogue no. GPQ-10, GCC Biotech) was used to calculate the total protein content of the supernatant using bovine serum albumin (BSA) as the reference. The amounts of each cytokine in the brain tissue were represented as pg/mg of protein [29].

### 2.15 Determination of protein expression of 5-HT2A and D2

For Western blot analysis, the cerebellum section was homogenised in RIPA (GCC Biotech, No. GLB01) buffer with protease (Catalogue: P8340 Sigma) and phosphatase inhibitors (GCC Biotech, GCR-P1), and the protein concentration was quantified by BCA assay. Total protein samples were separated by SDS-PAGE and transferred to PVDF membranes (IPVH00010, Millipore, USA). After being blocked with 3 % bovine serum powder for 1 h, the membranes were incubated with the primary antibody (5-HT2A No. MBS175200; MyBioSource, 1:1000; D2 ADI-905-740; Enzo Life Sciences, 1:250) overnight at 4 °C and the secondary antibody for 2 h at room temperature. Finally, the blots were developed by the ECL system (GCC Biotech, No. G7134), and bands visualised using the Simple Simon automatic Western BLOT system. β-Actin was used as a loading control. The blots were quantified using Image J software.

### 2.16 Statistical analysis

The data were analysed with SPSS Statistics version 19, and the data were represented as mean ± SEM. Statistical significance was determined using the one-way analysis of variance (ANOVA), followed by the Tukey HSD post hoc test. Significant levels of probability were p < 0.05; ns, non-significant (p > 0.05). Data expressed as mean ± SEM, n = 6. One-way ANOVA, followed by a post hoc Tukey's test. ^**a**^ p < 0.001, ^**b**^ p < 0.01, ^**c**^ p < 0.05 as compared to control; ^**α**^ p < 0.001, ^**β**^ p < 0.01, ^**γ**^ p < 0.05 as compared to VPA.

## 3 Results

### 3.1 In-vitro antioxidant assays

#### 3.1.1 Ferric reducing antioxidant power assay (FRAP)

Depending on the reducing power of these antioxidants, the colour of the testing solutions in the power reduction assay shifted from yellow to various shades of green and blue. The Fe3+/ferricyanide complex is reduced to the ferrous form when an antioxidant agent is present. In the present study, vitamin C at 0.25 mg/mL has a reducing power of OD value of 1.27 ± 0.004, EAFA at 0.25 mg/mL has a reducing power of OD value of 1.47 ± 0.343, and 70 % methanol extract at 0.25 mg/mL has a reducing power of OD value of 1.06 ± 0.008 as compared with vitamin C as a standard. Based on this finding, it appears that EAFA extract has more potent free radical scavenging activity than vitamin C. Furthermore, EAFA is more potent than 70 % methanol extract in Table S1. The results were expressed as the mean ± SEM of OD at 700 nm.

#### 3.1.2 Nitric oxide (NO) scavenging assay

The EAFA extract showed more potent radical scavenging activity on NO (IC50 = 0.082 ± 0.002) than the 70 % methanol extract (IC50 = 0.139 ± 0.002) compared to vitamin C as reference standards. The IC50 value of vitamin C is 0.004 ± 0.0002. This indicates EAFA has more potent radical scavenging activity than 70 % methanol extract because its IC50 is lower. The results of IC50 were expressed as mean ± SEM in mg/mL.

### 3.2 Qualitative analysis and identification of chemical constituents in EAFA using UPLC-ESI-Q-TOF-MS^E^ analysis

Based on information such as molecular ions, fragment ions, chemical formulas, and mass accuracy of less than 5 ppm acquired from UPLC-ESI-Q-TOF-MSE, chemical ingredients in EAFA were screened, and identified. Fig. S1 shows the base peak chromatogram of EAFA in both positive and negative modes. Using the ToF-MS and MS/MS spectra, we have found 47 different compounds. These include three amino acids, seven vitamins, two carbohydrates, twelve tannins, two flavonoids, three kaempferol derivatives, two quercetin derivatives, two flavones, one flavanone, two flavan-3-ols, six polyphenols, two other phenolics, one gallic acid derivative, one terpenoid, and one organic compound (Table S2).

### 3.3 UPLC-MS/MS quantification of phytochemical constituents in EAFA extract

The UPLC-MS/MS analysis of EAFA component quantification results is tabulated in Table S3. The analysis shows that ascorbic acid (vitamin C) was present at the highest concentration (69.29 ± 1.81) in the EAFA extract, followed by quercetin (7.88 ± 0.078), pantothenic acid gallic acid (2.72 ± 0.32), rutin (0.70 ± 0.029), and (0.07 ± 0.004). Chromatograms of phytoconstituents in EAFA extracts are represented in Fig. S2. The results of the quantification of phytoconstituents were expressed as mean ± SEM in μg/g of EAFA extract.

### 3.4 Effect of VPA on different developmental parameters

#### 3.4.1 Body weight changes and brain weight index

**Fig. 2:**
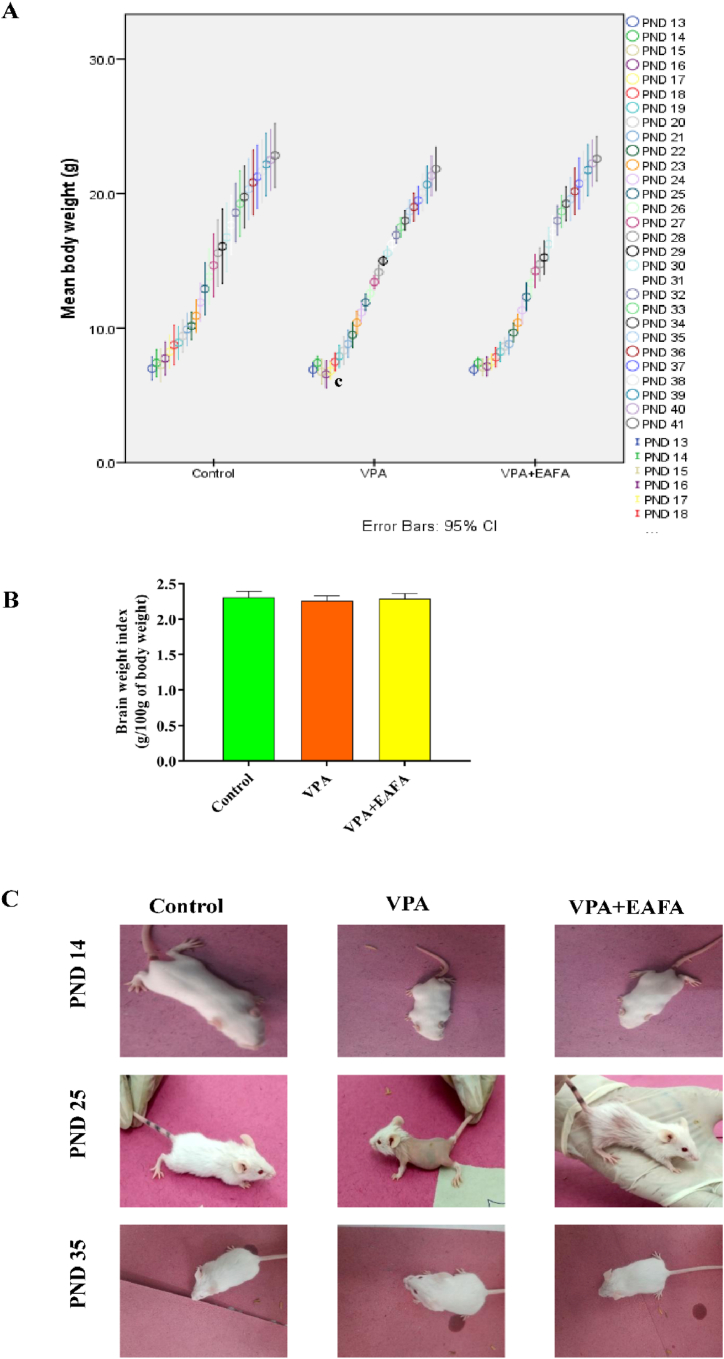
Effect of EAFA at 100 mg/kg dose on the developmental parameters of pups following administration of sodium valproate (VPA, 400 mg/kg s.c) on PND 14. **(A**) Body weight changes of each group were monitored daily; **(B)** brain weight index; (**C**) Each group's physical appearance representative hair coating images.

Sodium valproate (VPA, 400 mg/kg s.c.) was administered to PND-14 BALB/c mice, and the body weight resulted in no significant differences but a significant decrease (p < 0.024) in body weight on postnatal day 17 as compared to control mice. By day 18 and thereafter, no differences were observed in the VPA group. However, no differences in body weight were observed in the control and VPA + EAFA (100 mg/kg, BW orally) groups throughout the entire study (Fig. 2A). Apart from this, there were no significant differences in the brain-to-body mass ratio (g/100g of body weight) in any of the groups (Fig. 2B).

#### 3.4.2 Physical appearance

Upon the administration of 400 mg/kg of a single dose of VPA on PND 14 mice through the subcutaneous route, we found severe alopecia starting from PND 17 to PND 25 compared to control animals, whereas VPA + EAFA (100 mg/kg BW) treatment on VPA-treated mice showed mild alopecia compared to VPA-treated mice. Furthermore, from PND 28 onward, hair coating was observed in both groups, such as the VPA and VPA + EAFA groups, and animals that reached PND 35 showed restoration of the normal hair coat similar to the control group. However, control animals showed normal hair throughout the entire study (Fig. 2C).

### 3.5 Behavioural studies

#### 3.5.1 Effect of EAFA on motor coordination in VPA-postnatally exposed mice using a balance beam test

**Fig. 3:**
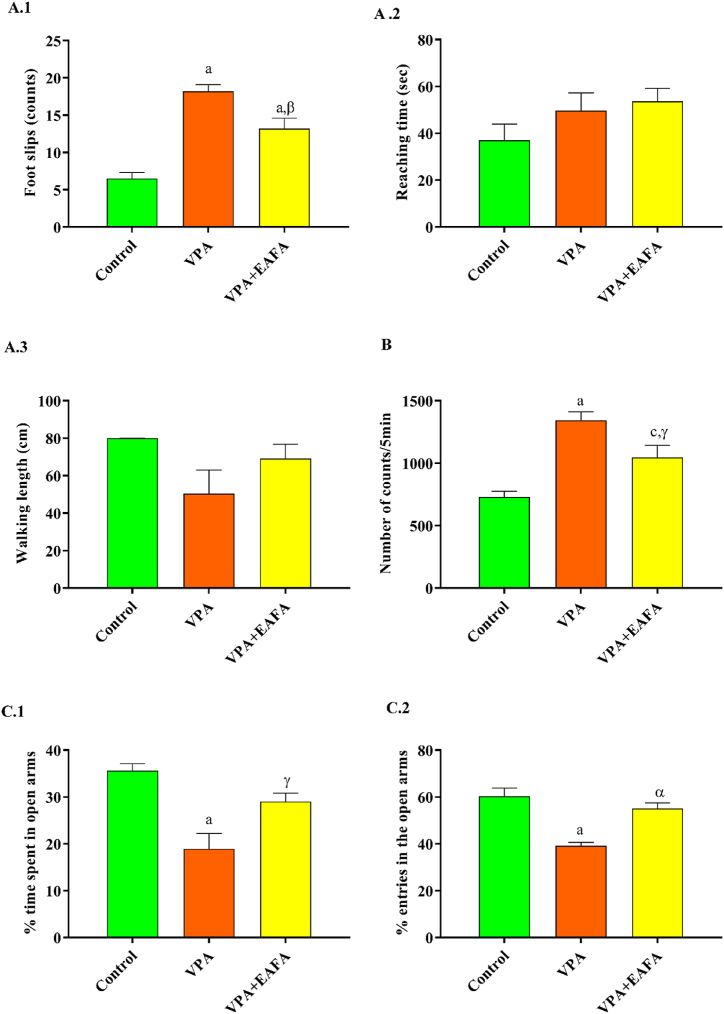
Effect of EAFA at 100 mg/kg doses on different behavioural parameters in sodium valproate-exposed postnatal BALB/c mice (VPA, 400 mg/kg, s.c) on PND 14. **(A)** Motor coordination ability was measured using the balance beam test on postnatal day 23–25: **(A.1)** Foot slips; **(A.2)** Reaching time (s); and **(A.3)** Walking length. **(B)** Locomotion due to hyperactivity measured on postnatal day 35. **(C)** Anxiety measured using the elevated plus maze apparatus on PND 40: (**C.1**) % Open arm time spent; **(C.2)** % Open arm entries.

A balance beam test was performed to assess motor coordination in experimental CNS disorders. The VPA-alone-treated mice showed significantly increased foot slips (p < 0.001; Fig. 3A.1) compared to the control. After EAFA administration orally (100 mg/kg) to VPA-exposed mice, the VPA-mediated foot slips significantly decreased (p < 0.01) compared with the VPA-alone-treated group. Thus, indicating a reduction in foot slips by the administration of EAFA to the VPA-exposed animals. However, no significant difference was observed in reaching time or walking length among all the groups. This effect was unchanged by VPA treatment (Fig. 3A.2–3).

#### 3.5.2 Effect of EAFA on locomotor activity using actophotometer in VPA-exposed mice

An actophotometer instrument was used to measure locomotion. When compared to control mice, locomotion in VPA-alone-treated mice increased significantly (p < 0.001). After EAFA administration (100 mg/kg BW) to VPA-exposed mice, the hyperactivity was significantly reduced (p < 0.03) compared with the VPA-alone-treated group. This indicates that locomotion incidence is reduced by the administration of EAFA to the VPA-exposed animals (Fig. 3B).

#### 3.5.3 Effect of EAFA on anxiety like behaviour on the elevated plus maze test in VPA-exposed mice

The percentage of time spent in the open arm (p < 0.001; Fig. 3C.1) and the percentage of open arm entries (p < 0.001; Fig. 3C.2) were significantly decreased in the postnatally exposed VPA-alone-treated group when compared with the control group. Treatment with EAFA to VPA-exposed mice has shown a significant increase in the% of time spent in the open arm (P < 0.02) and the% open arm entries (P < 0.001) when compared to the VPA-alone treated group and restored (p > 0.05 of those parameters) to control mice, thus demonstrating a reduction in anxiety by the administration of EAFA (100 mg/kg; orally) to the VPA-exposed animals.

### 3.6 Effect of EAFA on social affiliation in postnatal VPA-exposed mice

**Fig. 4:**
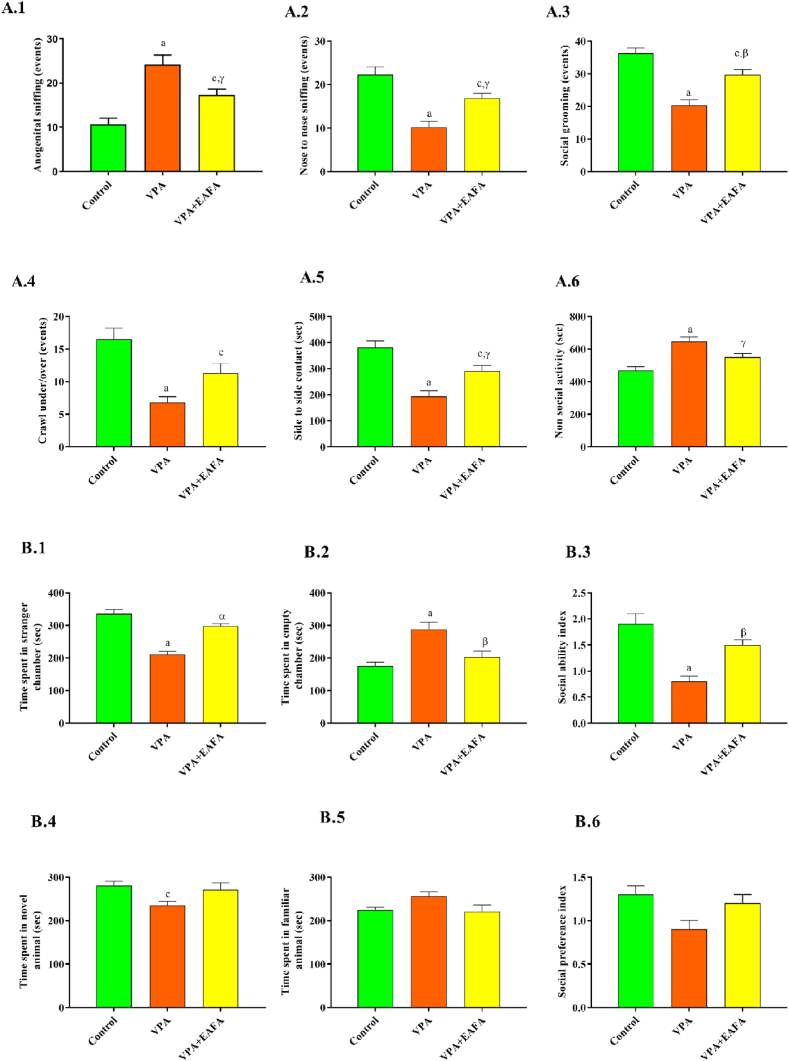
Effect of EAFA at 100 mg/kg doses on different social affiliation and interaction in sodium valproate-exposed postnatal BALB/c mice (VPA, 400 mg/kg, s.c) on PND 14. **(A)** Social affiliation response was measured by the duration of side-to-side contact with a cage mate on PND 35 to 40: **(A.1)** Anogenital sniffing; **(A.2)** Nose-to-nose sniffing; **(A.3)** Social grooming; **(A.4)** Crawl under/over; **(A.5)** Side-to-side contact (sec); **(A.6)** Non-social cage exploration (Rearing, ground exploration, self-grooming, air smelling and crouching in sec). **(B)** Social interaction test was conducted using a three-chamber apparatus on PND 35–40: (**B.1**) Time spent in stranger chamber; **(B.2)** Time spent in empty chamber; **(B.3)** Social ability index; **(B.4)** Time spent in novel chamber; **(B.5)** Time spent in familiar chambers; **(B.6)** Social preference index.

Adolescent affiliative behaviour was assessed with a cage-mate experiment. VPA-exposed mice significantly decreased the number of social explorations: nose-to-nose sniffing (p < 0.001), social grooming (p < 0.001), crawl under/over (p < 0.001), and side-by-side contact (p < 0.001), whereas they increased anogenital sniffing (p < 0.001) when compared to control mice. Treatment with EAFA in VPA-exposed mice has shown a significant (nose-to-nose sniffing p < 0.02; social grooming p < 0.004; side-by-side contact p < 0.02) increase in the number of social interactions except crawl under/over (p > 0.05), whereas there is a decrease in anogenital sniffing (p < 0.03) compared to the VPA-alone-treated group. Moreover, non-social cage exploration time was significantly (p < 0.001) increased in VPA-alone exposed mice compared to control mice, whereas treatment with EAFA in VPA-exposed mice has shown significant (p < 0.03) decreased non-social exploration compared to the VPA-treated group (Fig. 4A.1–4A.6). These findings suggest that EAFA administered orally at 100 mg/kg could restore social affiliations.

### 3.7 Effect of EAFA on social interaction in postnatal VPA-exposed mice

#### 3.7.1 Sociability and sociability index

In social experimental apparatus, control mice spent more time in the stranger chamber than in an empty chamber, indicating typical social behaviour. In contrast to control mice, postnatal VPA-alone-treated mice spent more time in the empty chamber (p < 0.001) and spent significantly less time in the stranger chamber (p < 0.001). According to this, postnatal VPA-alone-treated mice were not very social. In comparison to control mice, postnatal VPA-mice showed a significantly lower social index (p < 0.001). In addition, compared to postnatal VPA-alone-treated mice, VPA- and EAFA-treated mice spent more time in the stranger chamber (p < 0.001) and less time in the empty chamber (p < 0.01), and they had a higher social index (p < 0.003) (Fig. 4B.1–4B.3).

#### 3.7.2 Social preference and social preference index

During the social preference stage, control mice spent more time on the novel mouse as compared to the familiar mouse, which exhibits normal social preference behaviour. Postnatal VPA-alone treated mice exhibited significantly less time spent in the novel mouse (p < 0.05) and more time spent in the familiar chamber but not significantly (p > 0.05) as compared to control mice. This indicates the low social preference in postnatal VPA-alone-treated mice. Along with this, postnatal VPA-alone treated mice exhibited a reduction in social preference index (p > 0.05) but not significantly as compared to control mice. VPA + EAFA treated mice showed more time spent in the novel mouse (p > 0.05) and less time spent in the familiar mouse (p > 0.05); VPA + EAFA mice also showed an increment in the social preference index but not significantly (p > 0.05) as compared to postnatal VPA-alone treated mice (Fig. 4B.4- 4B.6).

### 3.8 Histopathology

**Fig. 5:**
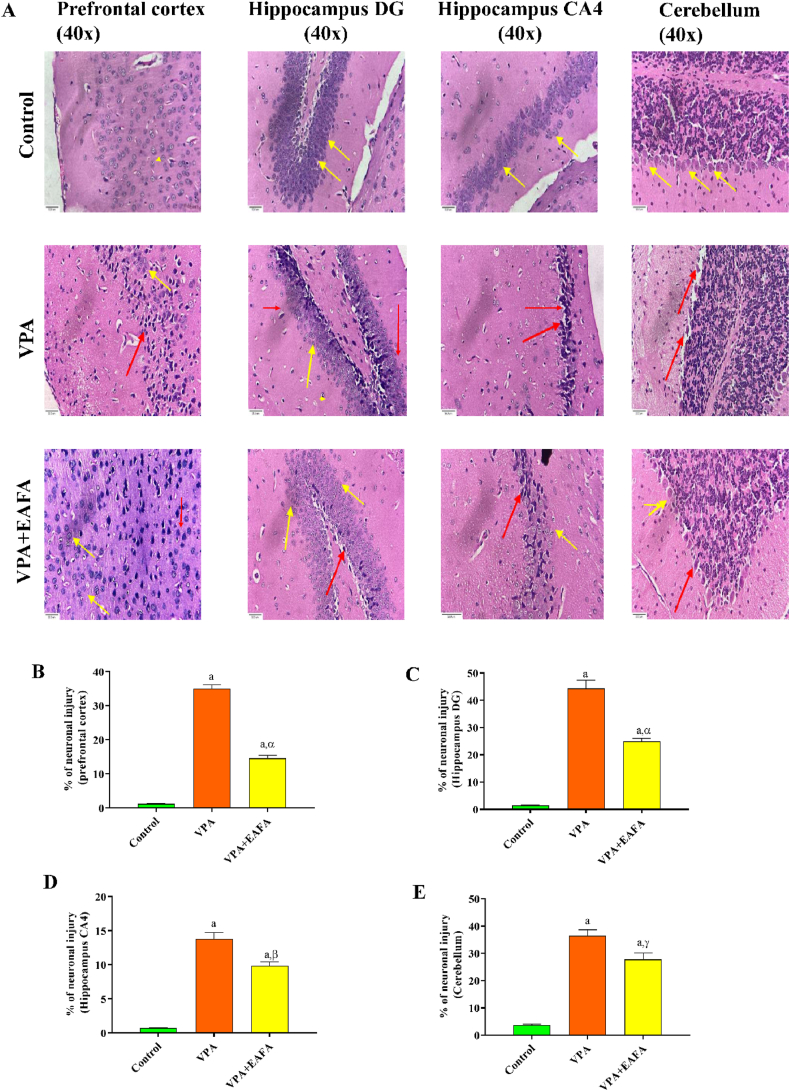
Effect of EAFA at 100 mg/kg doses on histopathological neuronal injury in sodium valproate-exposed postnatal BALB/c mice (VPA, 400 mg/kg, s.c.) on PND 14 (prefrontal cortex, hippocampus, and cerebellum). (**A**) Photomicrographs of H&E-stained sections of the prefrontal cortex, hippocampus (CA4 and DG), and cerebellum, (**B**) PFC neuronal injury score**,** (**C**) Hippocampus neuronal injury score of DG, (**D**) Hippocampus neuronal injury score of CA4, (**E**) Cerebellum neuronal injury score. Scale bar: 30 μm. Final magnification 40**×**. Red arrows indicate injured neurons and yellow arrows indicate normal neurons. (For interpretation of the references to colour in this figure legend, the reader is referred to the Web version of this article.)

The histological examination using H and E staining showed that, in contrast to the control group, the VPA-alone-treated group hippocampus showed severe neuronal damage in the CA4 and dentate gyrus (DG) areas, which was evident in the form of shrunken and pyknotic nuclei in the neurons of those regions. Additionally, similar pyknotic neurons were present in the PFC regions of the VPA-alone-treated group, indicating neuronal injury in these areas. Furthermore, mice with VPA treatment exhibited atrophic Purkinje cells in their brains (Fig. 5A–E).

The percentage of neuronal injury was significantly higher in the VPA-alone-exposed animals than in the control group (p < 0.001), while treatment with EAFA at a dose of 100 mg/kg considerably reduced the neuronal injury in CA4 (p < 0.002) and dentate gyrus (p < 0.001) as compared to the VPA-alone-treated group. In addition, the VPA-alone-treated group prefrontal cortex neuronal damage percentage was substantially higher (p < 0.001) than that of the control group. Thus, EAFA at 100 mg/kg treated VPA-exposed mice considerably reduced (p < 0.001) neuronal damage compared to the VPA-alone-treated group. Furthermore, a significant difference in atrophic cerebellar Purkinje cells was observed in VPA-treated animals (p < 0.001) compared to the control group, whereas treatment with EAFA at a dose of 100 mg/kg significantly reduced the percentage of neuronal injury in the cerebellum (p < 0.01) compared to the VPA-alone-treated group (Fig. 5B-E).

### 3.9 Scanning electron microscope analysis

**Fig. 6:**
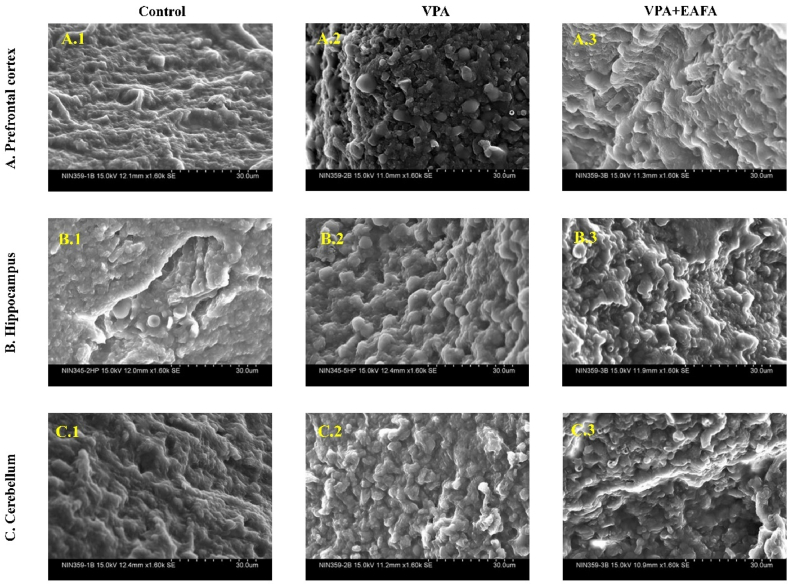
Effect of EAFA at 100 mg/kg doses on cellular morphology changes in sodium valproate-exposed postnatal BALB/c mice (VPA, 400 mg/kg, s.c) on PND 14 by scanning electron microscopy analysis in mouse brains of the (**A**) Prefrontal cortex (A.1-A.3), (**B**) hippocampus (B.1-B.3), and (**C**) cerebellum (C.1-C.3), n = 3, scale bar: 30 μm. Final magnification: 1.60 k × .

The morphological features of the brains of the control, VPA-alone group, and VPA + EAFA groups were assessed using scanning electron microscopy (Fig. 6). In our study, three brain regions were selected for SEM analysis: A higher rate of ultrastructural changes in the peripheral region of the prefrontal cortex (Fig. 6.A1-A3), the hippocampus (Fig. 6.B1–B3), and the cerebellum (Fig. 6.C1–C3) were observed in the VPA-alone group compared to the control group. Treatment with EAFA at a 100 mg/kg dose showed ameliorated VPA-induced cellular structural alterations in the peripheral region of the prefrontal cortex, hippocampus, and cerebellum compared to the VPA-treated group, whereas control animals showed normal cellular structure.

### 3.10 Immunohistochemical studies

#### 3.10.1 Effect of EAFA on serotonin (5-HT1D and 5-HT2A) receptor proteins in postnatal VPA-exposed mice

**Fig. 7:**
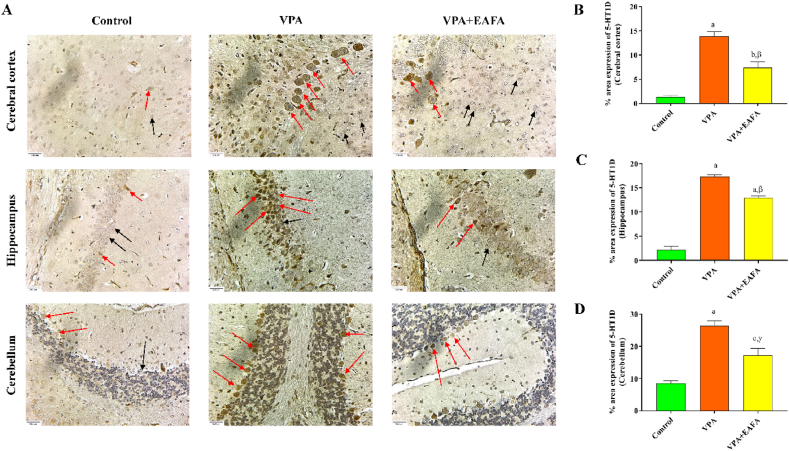
Effect of EAFA at 100 mg/kg doses on IHC immunoreactivity of 5-HT1D receptor proteins in sodium valproate-exposed postnatal BALB/c mice (VPA, 400 mg/kg, s.c) on PND 14 in the mouse (**B**) brain's cerebral cortex, (**C**) hippocampus and (**D**) cerebellum. The positive cells were stained in brown (Red arrows), and black arrows indicate normal neurons (**A**). Relative protein expression in % of 5-HT1D was quantified by Fiji (Image J) software tools. Scale bar:30 μm. Final magnification 40 × . (For interpretation of the references to colour in this figure legend, the reader is referred to the Web version of this article.)

**Fig. 8:**
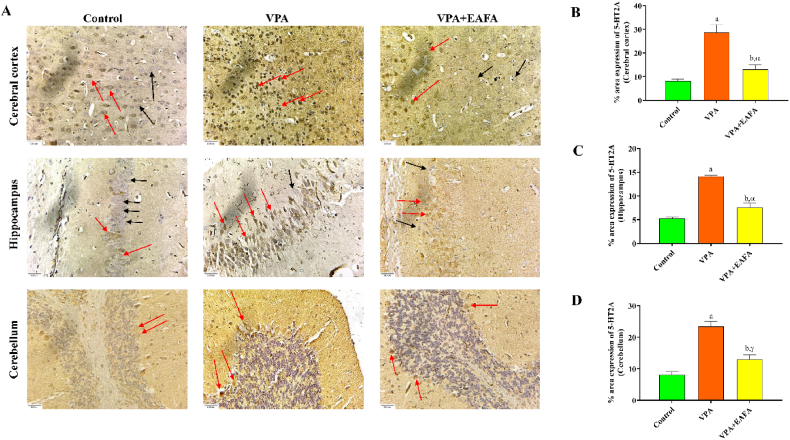
Effect of EAFA at 100 mg/kg doses on IHC immunoreactivity of 5-HT2A receptor proteins in sodium valproate-exposed postnatal BALB/c mice (VPA, 400 mg/kg, s.c) on PND 14 in the mouse (**B**) brain's cerebral cortex, (**C**) hippocampus and (**D**) cerebellum. The positive cells were stained in brown (Red arrows), and black arrows indicate normal neurons (**A**). Relative protein expression in % of 5-HT2A was quantified by Fiji (Image J) software tools. Scale bar: 30 μm. Final magnification 40 × . (For interpretation of the references to colour in this figure legend, the reader is referred to the Web version of this article.)

In the current study, we first observed the levels of 5-HT1D and 5-HT2A receptor protein expressions in the postnatal mice treated with VPA by immunohistochemical analysis. Compared to control animals, VPA-alone-treated mice had more positive expression of 5-HT1D (p < 0.001 in Fig. 7) and 5-HT2A (p < 0.001 in Fig. 8), receptor proteins in the Purkinje and granular cells of the cerebellum, cerebral cortex, and hippocampus. Furthermore, treatment with EAFA on VPA-exposed mice showed expression of these serotonin receptor proteins was significantly decreased in the Purkinje and granular cells of the cerebellum (p < 0.017 in Fig. 7D; p < 0.039 in Fig. 8D), cerebral cortex (p < 0.005 in Fig. 7B; p < 0.001 in Fig. 8B), and hippocampus (p < 0.003 in Fig. 7C; p < 0.001 in Fig. 8C) compared to VPA-alone-treated mice. These findings indicate that EAFA treatment (100 mg/kg, orally) given to VPA-exposed mice could attenuate the 5-HT1D (Fig. 7A) and 5-HT2A (Fig. 8A) receptor proteins.

### 3.11 Biochemical assessments in prefrontal cortex and cerebellum homogenates

**Fig. 9:**
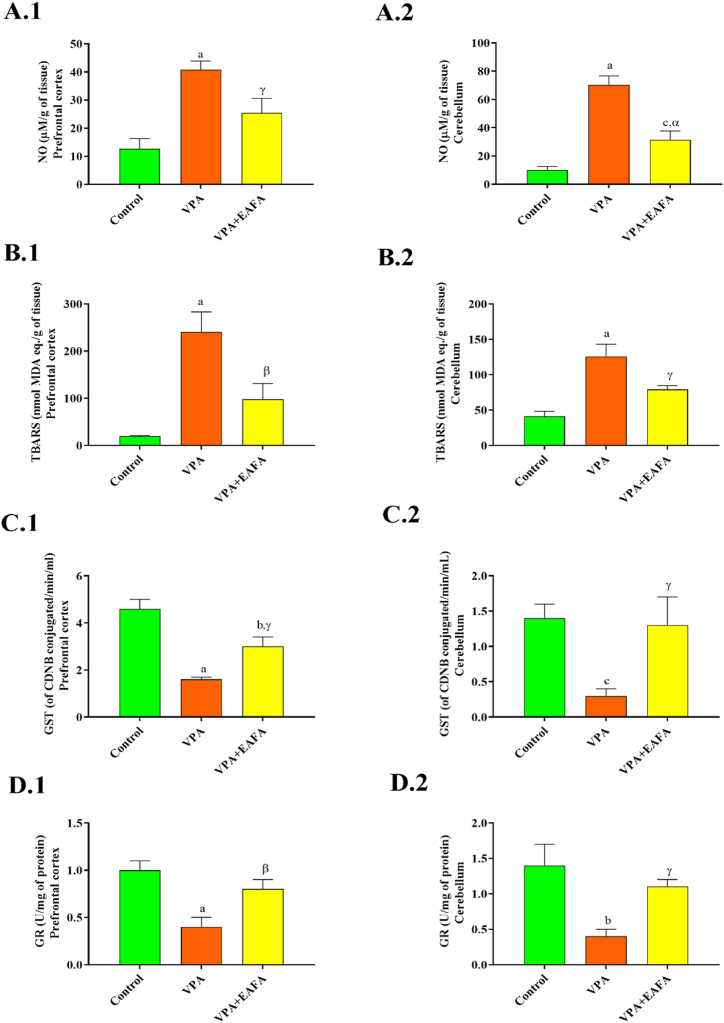
Effect of EAFA at 100 mg/kg doses on inflammatory and oxidative stress in the PFC and cerebellum of sodium valproate-exposed postnatal BALB/c mice (VPA, 400 mg/kg, s.c) on PND 14, (**A.1, A.2**) NO, (**B.1, B.2**) MDA, (**C.1, C.2**) GST, (**D.1, D.2**) GR.

As compared to control mice, postnatal VPA-alone-treated mice had significantly higher MDA (p < 001 Fig. 9B.1; p < 001 Fig. 9B.2) and NO (p < 001 Fig. 9A.1; p < 001 Fig. 9A.2) levels, as well as lower glutathione-related enzymes such as GST (p < 001 Fig. 9C.1; p < 001 Fig. 9C.2) and GR (p < 001 Fig. 9D.1; p < 001 Fig. 9D.2) levels in the prefrontal cortex and cerebellum, respectively, indicating oxidative stress. When compared to postnatal VPA-alone-treated mice, VPA + EAFA mice showed significantly lower MDA (p < 0.01; p < 0.03) and NO (p < 0.04; p < 0.001) and higher GST (p < 0.03; p < 0.04) and GR (p < 0.002; p < 0.04) levels in the prefrontal cortex and cerebellum, respectively, in Fig. 9A–D. Thus, it indicates a reduction in oxidative and nitrosative stress markers by the administration of EAFA to the VPA-exposed mice as compared to postnatal VPA-alone-treated mice. Therefore, EAFA extracts have a high potential for anti-oxidant activity and might reduce oxidative stress-mediated neuroinflammation (Fig. 9).

### 3.12 Estimation of pro-inflammatory cytokines

**Fig. 10:**
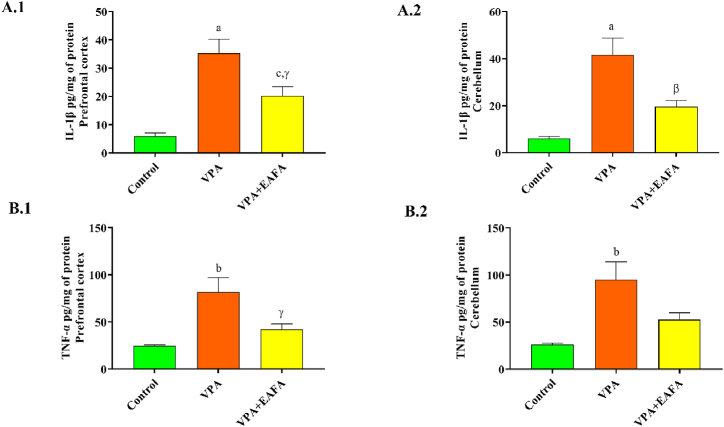
Effect of EAFA at 100 mg/kg doses on proinflammatory in the PFC and cerebellum of sodium valproate-exposed postnatal BALB/c mice (VPA, 400 mg/kg, s.c) on PND 14, (**A.1**) IL-1β in the PFC, (**A.2**) IL-1β in the cerebellum, (**B.1**) TNF-α in the PFC, (**B.2**) TNF-α in the cerebellum.

The levels of IL-1β (Fig. 10A.1–2) and TNF-α (Fig. 10B.1–2) were measured in both the prefrontal cortex and cerebellum using the ELISA assay. IL-1β (p < 0.001; p < 0.001) and TNF-α (p < 0.002; p < 0.002) levels were markedly increased in the VPA group in comparison to the control in both the prefrontal cortex and cerebellum. Treatment with EAFA at a 100 mg/kg dose in VPA-exposed animals significantly decreased the IL-1β in the cerebellum (p < 0.008) and prefrontal cortex (p < 0.02), but TNF-α levels significantly decreased in the prefrontal cortex (p < 0.02) but not in the cerebellum (p > 0.05) compared to the VPA group (Fig. 10).

### 3.13 Western blot analysis

#### 3.13.1 Effect of EAFA on 5-HT2A in the cerebellum of postnatally VPA-exposed mice

**Fig. 11:**
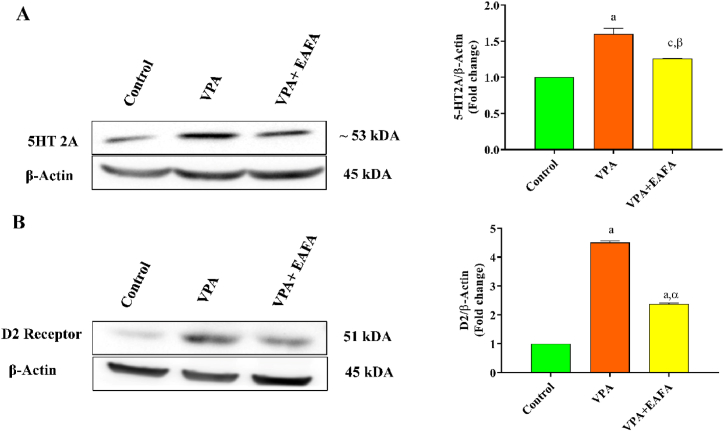
Effect of EAFA at 100 mg/kg doses on the protein expression of cerebellum (**A**) 5-HT2A and (**B**) D2 by Western blot in postnatally VPA exposed mice (VPA, 400 mg/kg, s.c) on PND 14.

We first observed that postnatal mice exposed to VPA alone showed a significant increase in the level of 5-HT2A (p < 0.001 in Fig. 11A) receptor protein expression in the cerebellum compared to the control group. Furthermore, EAFA at 100 mg/kg treatment in VPA-exposed mice showed that 5-HT2A levels significantly decreased (p < 0.007) compared to VPA-alone-treated mice (Fig. 11A). According to IHC analysis in this experiment, we have seen a similar result pattern, which is quite similar to the western blotting analysis.

#### 3.13.2 Effect of EAFA on the D2 receptor in the cerebellum of postnatally VPA-exposed mice

Dopamine (DA) is a key neurotransmitter involved in social behaviour, social cognition, and movement regulation. Compared to the control mice, postnatal VPA-alone-exposed mice showed significantly increased levels of D2 receptor protein in the cerebellum (p < 0.001 in Fig. 11B), whereas treatment with EAFA at a dose of 100 mg/kg to VPA-exposed mice significantly decreased the levels of D2 receptor protein (p < 0.001) compared to the VPA-alone-treated group (Fig. 11B).

## 4 Discussion

Oxidative stress develops when the body produces more reactive oxygen/nitrogen species and its antioxidant defences are less effective. The generation of reactive oxygen/nitrogen species (ROS/RNS) causes a loss of antioxidant capacity [31]. This oxidative stress was associated with neurodevelopmental disorders like ASD [32]. The fruit of *Phyllanthus emblica* was edible and rich in flavonoids, tannins, polyphenols, and other substances with strong antioxidant properties. As a result, it can be added to food for general consumption as well as used as a supplement to improve human health [23]. In the present study, the ferric-reducing antioxidant power test (FRAP test) was used to determine the total antioxidant activity of 70 % methanol amla extract and the ethyl acetate fraction of amla extract (EAFA). An increase in reducing power is shown by increases in absorbance at this wavelength. EAFA had a greater reduction power than 70 % methanol amla extract in the current investigation. Additionally, the Griess reagent was used to assess the free radical NO scavenging capacity of 70 % methanol amla extract and EAFA extract. As a result, the EAFA extract and 70 % methanol amla extract, which scavenge free radicals in NO, may effectively inhibit reactive nitrogen stress. The EAFA fraction has the greatest capacity to scavenge free radicals in NO compared to 70 % methanol amla extract. According to our earlier reported study, the ethyl acetate fraction of amla (EAFA) extract showed more polyphenol and antioxidant potential than 70 % methanol amla extract [18]. Hence, we selected the EAFA extract to treat the animals in the current study.

The present study demonstrated that EAFA ameliorated the behavioural abnormalities of VPA exposure, as indicated by increased sociability and social affiliative behaviour, a decrease in foot slips and locomotion activity, and an improvement in anxiety-like behaviour. It also reduced neuroinflammation as well as decreased oxidative stress in the prefrontal cortex and cerebellum in the pups of VPA-exposed animals or VPA + EAFA-treated animals. Additionally, EAFA at a dose of 100 mg/kg attenuated up-regulated 5-HT2A and D2 receptor expression in the cerebellum and reversed the neuronal injury in the prefrontal cortex, hippocampus, and cerebellum.

In this study, based on a physical examination, animals were exposed to VPA alone at 400 mg/kg subcutaneously. We have observed that the hair loss starts with PND17 in mice. Moreover, according to our previous study and this study, VPA-treated mice showed hair loss from PND 17 to 25, which might be due to a biotin deficiency [11], whereas VPA-exposed animals treated with EAFA showed mild hair loss, which might be due to a biotin present in amla. But starting at PND 28, hair started to grow on both the VPA-alone group and the EAFA-treated groups. By the time the animals reached PND 35, they had a regular hair coat like control animals.

Numerous physiological processes, including psychoemotional, autonomic, sensory, and motor processes, were controlled by the serotonergic system [33]. Diverse 5-HT receptors are involved in mediating serotonergic neurotransmission. According to previous reports, 5-HT2A and 5-HT1D receptors were critical in certain CNS illnesses, such as ASD (Gouda et al., 2022).

In the present study, postnatal VPA-alone treated mice led to a marked increase in the level of 5-HT2A in the cerebral cortex, hippocampus, and cerebellum by IHC analysis. Furthermore, the expression of 5-HT2A receptor protein increased in the cerebellum, which was also investigated by Western blot analysis and was quite similar to the results of immunohistochemical analysis. However, the results of the current study showed a significant decrease in the protein expression of 5-HT2A receptors on treatment with EAFA at a 100 mg/kg dose compared to VPA-alone exposed mice.

According to previous reports, motor incoordination [32] and social anxiety [34,35] were also recorded for ASD patients. The 5-HT2A receptor plays a significant role in regulating anxiety-related behavior [36,37] and motor coordination [38] because the 5-HT2A receptor's activation might lower 5-HT outflow and lower serotonergic neuron activity. In the present study, VPA-alone-treated mice showed an increase in foot slips (loss of motor coordination), which was assessed by conducting a balance beam test, as well as increased anxiety, which was assessed by conducting the elevated plus maze experiment. According to the aforementioned studies and the current study, VPA animals showed elevated levels of the 5-HT2A receptor protein in the cerebellum, which may have increased anxiety [36,37] and loss of motor coordination [38]. After treatment with EAFA at a dose of 100 mg/kg BW in VPA-exposed animals, the protein expression of the 5-HT2A receptor significantly decreased, which may have led to a significantly decreased foot slip and reduced anxiety.

Persistent difficulties in social communication and social interaction were important characteristics of ASD, according to the DSM-5 diagnostic criteria [13]. In line with what had been found before in the three chamber test, VPA-exposed mice in our study showed significantly low sociability, sociability index, and social preference, but not social preference index, compared to control mice [2,11], whereas the EAFA-treated group improved the social interactions compared to the VPA-alone group.

Along with this, an important factor in the detection and diagnosis of ASD was social affiliation behaviour [39]. Hence, social affiliation was assessed by conducting a cage-mate experiment on VPA-induced autism [26]. In the present study, postnatally exposed VPA mice showed reduced social affiliation and elevated levels of non-affiliative behaviours with a same-sex family, whereas treatment with the EAFA group significantly improved those social affiliation parameters and reduced the non-affiliative behaviours. Even though the pathogenesis of social deficits in ASD is not clear, one of the hypotheses was dysfunction of excitatory/inhibitory signalling in the ASD brain [40]. The upregulation of glutamatergic receptor activity in the brain may be responsible for the social behaviour abnormalities in VPA-alone exposed animals [41]. A study by Schiavi et al. (2019) [13] and Cutando et al. (2022) [42] also revealed that D2 receptor signalling modulated social behaviour. In evidence from the aforementioned studies and the current study, it was revealed that elevated D2 receptor expression causes decreased social interaction and social affiliation behaviour in VPA-alone-exposed mice. Whereas the EAFA-treated group showed diminished D2 receptor expression, which helps to improve social activities and affiliation behaviours.

Furthermore, hyperlocomotion was one of the symptoms of ASD [43]. According to our study's findings, the VPA-alone group had more locomotor behaviour in novel environments, which is consistent with other studies [5,25]. This may be due to brain anomalies induced by VPA or increased glutaminergic transmission leading to neural hyperexcitability in brain regions [44,45]. In many brain areas, there was an inverse relationship between glutamate and dopamine synthesis [46]. According to this study, elevated D2 receptor protein expression in the cerebellum of VPA animals may result in elevated glutaminergic transmission, leading to neural hyperexcitability in brain regions, whereas the EAFA-treated group revealed that it reduced the D2 receptor protein expression, leading to normalised locomotor activity.

There was evidence that the cerebellum, hippocampus, and prefrontal cortex play a substantial role in autism and the development of autistic characteristics in response to VPA [35,47,48]. The cerebellum has a role in sociability and motor functions; the hippocampus was involved in memory and learning; and the prefrontal cortex has a role in anxiety, social, and exploratory behaviour [14]. Based on histopathological evidence, similar to previous studies and our study, postnatal VPA-exposed mice showed signs of apoptosis in the prefrontal cortex, dentate gyrus, and Cornu ammonis 4 (CA4) region of the hippocampus [32]. These signs were detected by using H&E staining under light microscopy, in which degenerating neuronal cells appeared darkly stained with pyknotic nuclei. As a result, damage to the Purkinje cells prevents information from properly transferring from the cerebellum to the cerebral cortex, which may lead to autism like symptoms in the VPA-induced autism animal model. Whereas the EAFA-treated group showed significant protection against the VPA-induced neuronal injury. Aside from this, in the current study, detailed morphological observations using scanning electron microscopy (SEM) showed that VPA animals' prefrontal cortex, hippocampus, and cerebellum had a higher rate of structural changes in the outer part of these areas. This structural change could be one of the causes of these animals' abnormal behaviour. Treatment with EAFA at 100 mg/kg BW reduced postnatal VPA-induced cellular structural changes.

One of the main causes of ASD was thought to be the oxidative stress response [49,50], inflammation and neurodevelopmental problems have both been linked to high NO production in earlier studies [31]. Due to their low plasma and cellular glutathione levels, ASD patients are thought to be more susceptible to oxidative damage. According to the current study, the VPA-alone group showed a significantly lower amount of both antioxidant enzymes such as GR and GST as well as increased MDA and NO levels compared to the control group in the PFC and cerebellum tissue. The findings of this investigation on the EAFA-treated group showed significantly restored levels of those antioxidant enzymes, such as GR and GST, and decreased MDA and NO levels. Hence, the EAFA-treated group reduced the oxidative stress brought on by VPA. In numerous CNS illnesses, such as Alzheimer's disease [22], the antioxidant activity of amla has already been documented. Therefore, EAFA extract acts as a potent antioxidant agent.

Numerous studies have shown that people with ASD have higher levels of pro-inflammatory cytokines like IL-1β and TNF-α [51]. The results of the current experiment further emphasised the potential role of inflammation in ASD caused by VPA. The various studies indicate that cytokines are critical for the normal neurodevelopmental process and that any abnormalities in cytokines can impair neurons' ability to develop normally. In the current investigation, PFC and the cerebellum of the VPA-alone group showed higher levels of pro-inflammatory cytokines such as IL-1β and TNF-α compared to the control group. The findings of this investigation showed that EAFA significantly attenuates VPA-induced pro-inflammatory cytokines. These findings are consistent with earlier research, which showed that long-term amla administration significantly attenuated the pro-inflammatory cytokines [52,53]. Therefore, EAFA extract acts as an anti-inflammatory agent.

According to our earlier research, the EAFA extract contains a high concentration of polyphenols [18] that can pass the blood-brain barrier and potentially counteract the effects of valproate by inducing neuronal protection [25]. In the present study, five major compounds in the EAFA extract were quantified by using the UPLC-MS/MS analytical technique. According to previous reports, some of the active constituents, such as quercetin [54], rutin [55], pantothenic acid [56], gallic acid [57], and ascorbic acid [58] were well-known compounds for neuroprotective activity. Our quantitative analysis suggests these five active constituent combinations were found in the EAFA extract, which could significantly restore the antioxidant defence mechanism through antioxidant potential activity and reduce the dopaminergic and serotonergic levels against VPA-induced autism. Hence, EAFA is suggested as a potent neuroprotective agent.

In summary, our study showed that the EAFA extract would be a good candidate for reducing the behavioural abnormalities of VPA-induced postnatal autism in mice. The underlying mechanisms may be downregulation of 5-HT2A and D2 receptor protein expression, upregulation of the antioxidants GST and GR, and reduction of brain oxidative stress, inflammation, and apoptosis. These changes lead to normalising serotonergic and dopaminergic transmission, which may minimise autism symptoms.

## 5 Conclusion

In this conclusion, our current study suggests that treatment with EAFA extract relieved the VPA-induced autism due to its anti-oxidant, anti-inflammatory, and neuroprotective properties and supports the clinical feasibility of its use in treating autism-like conditions in humans.

## Ethics statement

All experimental protocols for the management and supervision of animal experiments were carried out in compliance with the CPCSEA guidelines. The Institutional Animal Ethics Committee reviewed and approved this study (ICMR-NIN/IAEC/02/007/2019, ICMR-NIN Animal Facility, National Institute of Nutrition, Hyderabad, India).

## Funding

The authors are thankful to the 10.13039/501100001411Indian Council of Medical Research (10.13039/501100001411ICMR) for granting the fellowship (3/1/2/62/2015-Nut) and to the 10.13039/501100024390ICMR-National Institute of Nutrition and 10.13039/501100004207Osmania University for providing the necessary infrastructure, encouragement, and support.

## Informed consent statement

Not applicable.

## Data availability statement

The data that support the findings of this study are available from the corresponding author, upon reasonable request.

## CRediT authorship contribution statement

**Balaji Gouda:** Conceptualization; project administration; experimentation; validation; methodology; formal analysis; writing—original draft. **Sukesh Narayan Sinha:** Supervision; resources; project administration; methodology; investigation; formal analysis; validation; review. **Rajendra Sangaraju:** Formal analysis; experimentation; validation; writing-review and editing. **Tien Huynh:** Writing—review and editing; formal analysis. **Patangay Shashikala:** Supervision; project administration; methodology; investigation; formal analysis; validation; review. **Venkata Mullapudi Surekha:** formal analysis; review and editing. **Sathish Kumar Mungamuri:** Review. **Pradeep B. Patil:** Formal analysis. **Madhusudhana Chary Periketi:** Formal analysis.

## Declaration of competing interest

The authors declare the following financial interests/personal relationships which may be considered as potential competing interests: Dr. Sukesh Narayan Sinha reports financial support was provided by 10.13039/501100001411ICMR - 10.13039/501100024390National Institute of Nutrition. Dr. Sukesh Narayan Sinha reports a relationship with 10.13039/501100001411ICMR - 10.13039/501100024390National Institute of Nutrition that includes: funding grants. If there are other authors, they declare that they have no known competing financial interests or personal relationships that could have appeared to influence the work reported in this paper.
