# Supplementary material for: Extraction, Phytochemical profile, and neuroprotective activity of Phyllanthus emblica fruit extract against sodium valproate-induced postnatal autism in BALB/c mice
Source: Heliyon. 2024 Jul 20;10(15):e34992. doi: 10.1016/j.heliyon.2024.e34992 (PMC11327600; doi:10.1016/j.heliyon.2024.e34992)
Supplement: Multimedia component 1 [file mmc1.docx]

Supplementary Material

**Extraction, Phytochemical Profile and Neuroprotective Activity of *Phyllanthus emblica* Fruit Extract Against Sodium Valproate-Induced Postnatal Autism in BALB/c Mice**

Fig. S1 MS chromatogram of EAFA extract


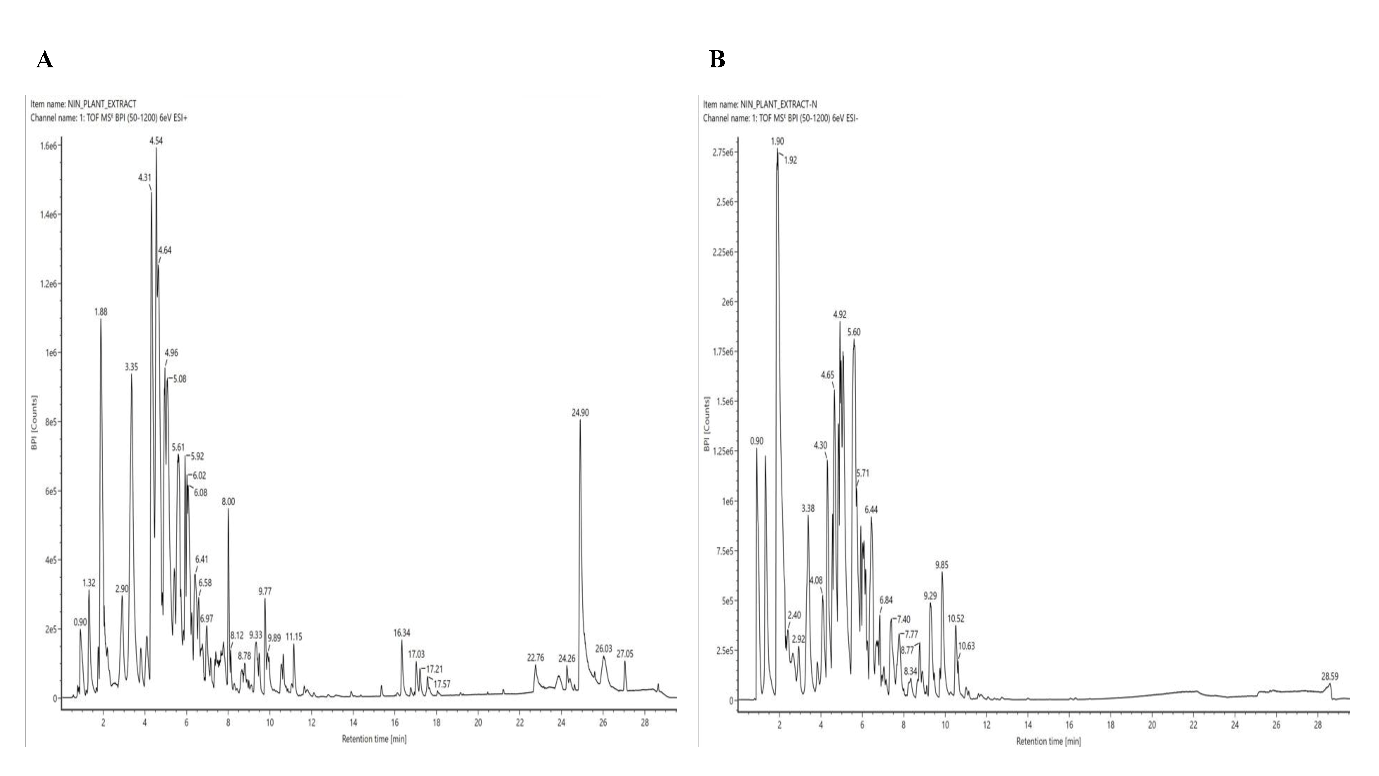


**Fig. S2** The quantification of compound chromatograms in an EAFA extract sample using UPLC-MS/MS. (**A**) Ascorbic acid and Gallic acid; (**B**) Quercetin and Rutin; (**C**) pantothenic acid.

**
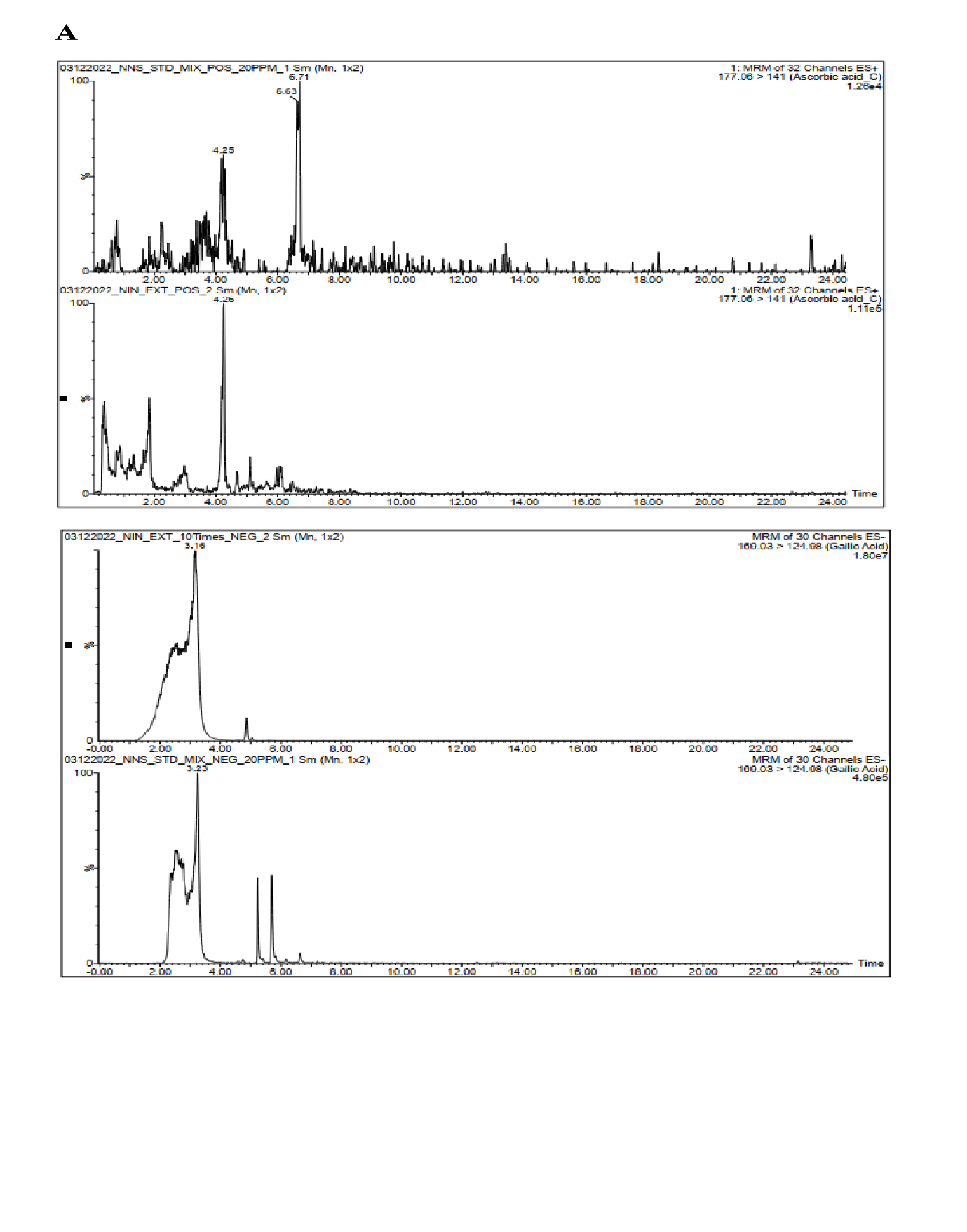
**

**
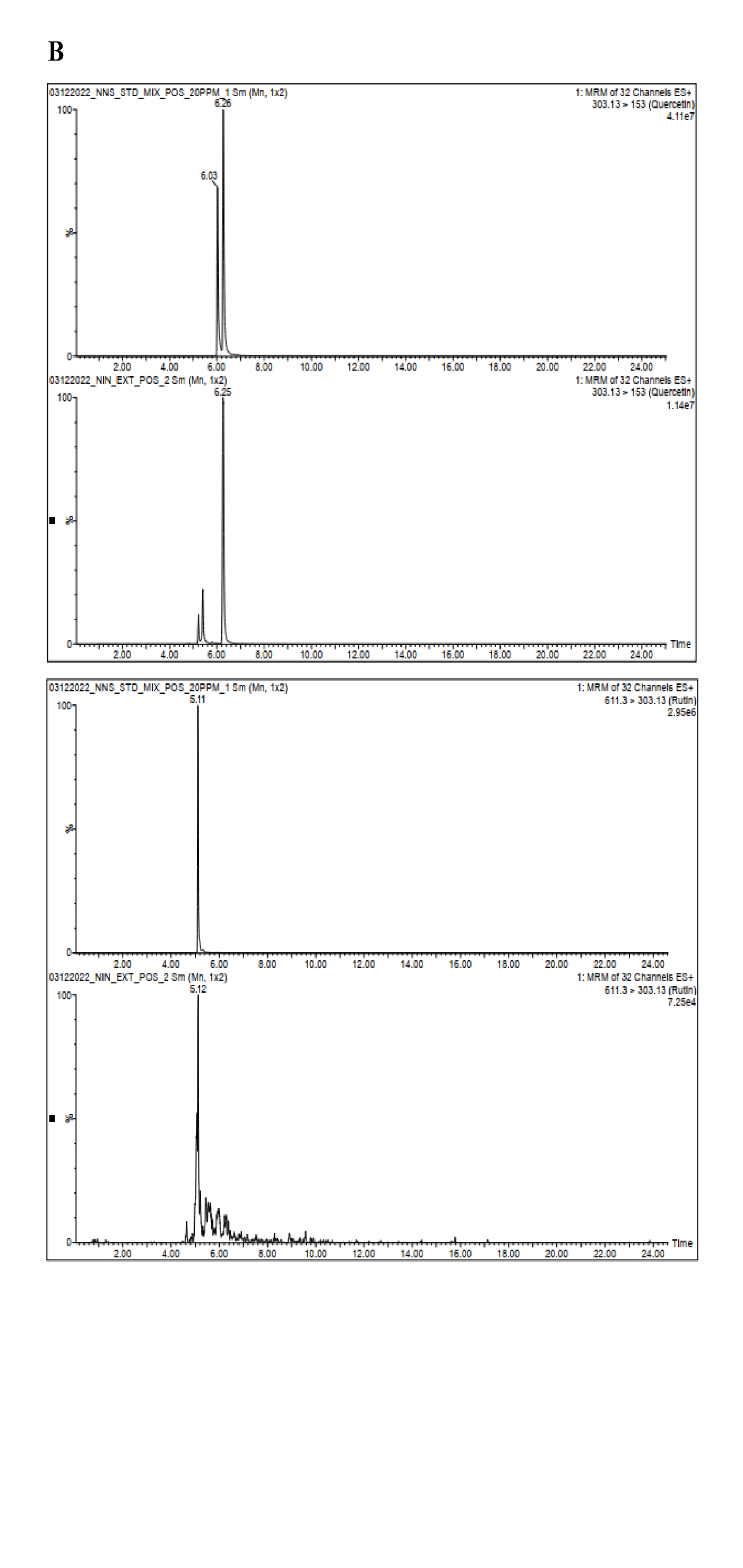
**

**
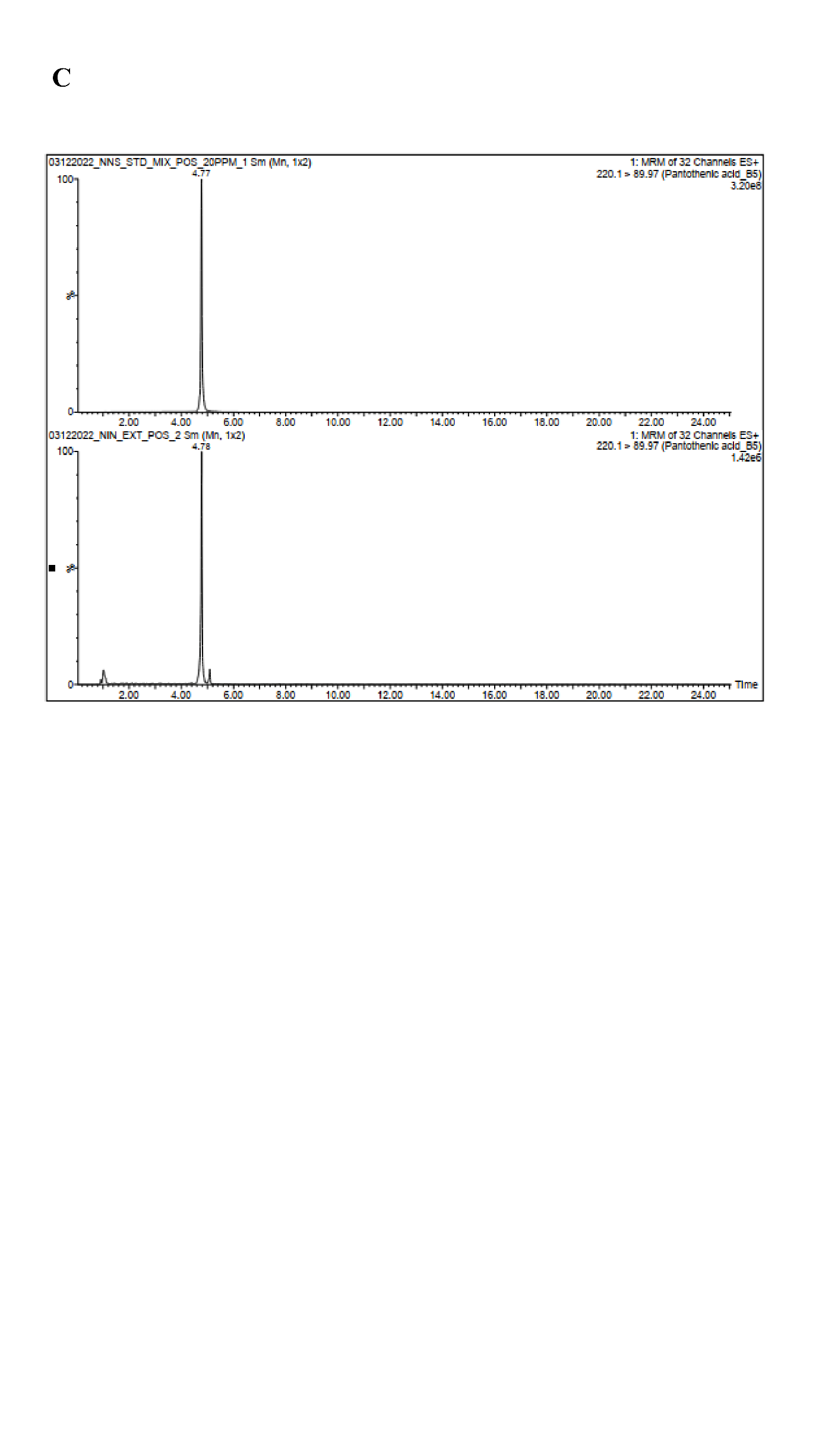
**

**Fig. S3** Effect of EAFA at 100 mg/kg doses on the protein expression of (**A**) 5-HT2A, and (**B**) D2 by western blot in postnatally VPA exposed mice (VPA, 400 mg/kg, s.c) on PND 14 in the cerebellum. Data expressed as mean ± SEM (n = 3). A one-way ANOVA was used, followed by Tukey’s post hoc test. **^a^** p < 0.001, **^b^** p < 0.01, **^c^** p < 0.05 as compared to control; **^α^** p < 0.001, **^β^** p < 0.01, **^γ^** p < 0.05 as compared to VPA.

**
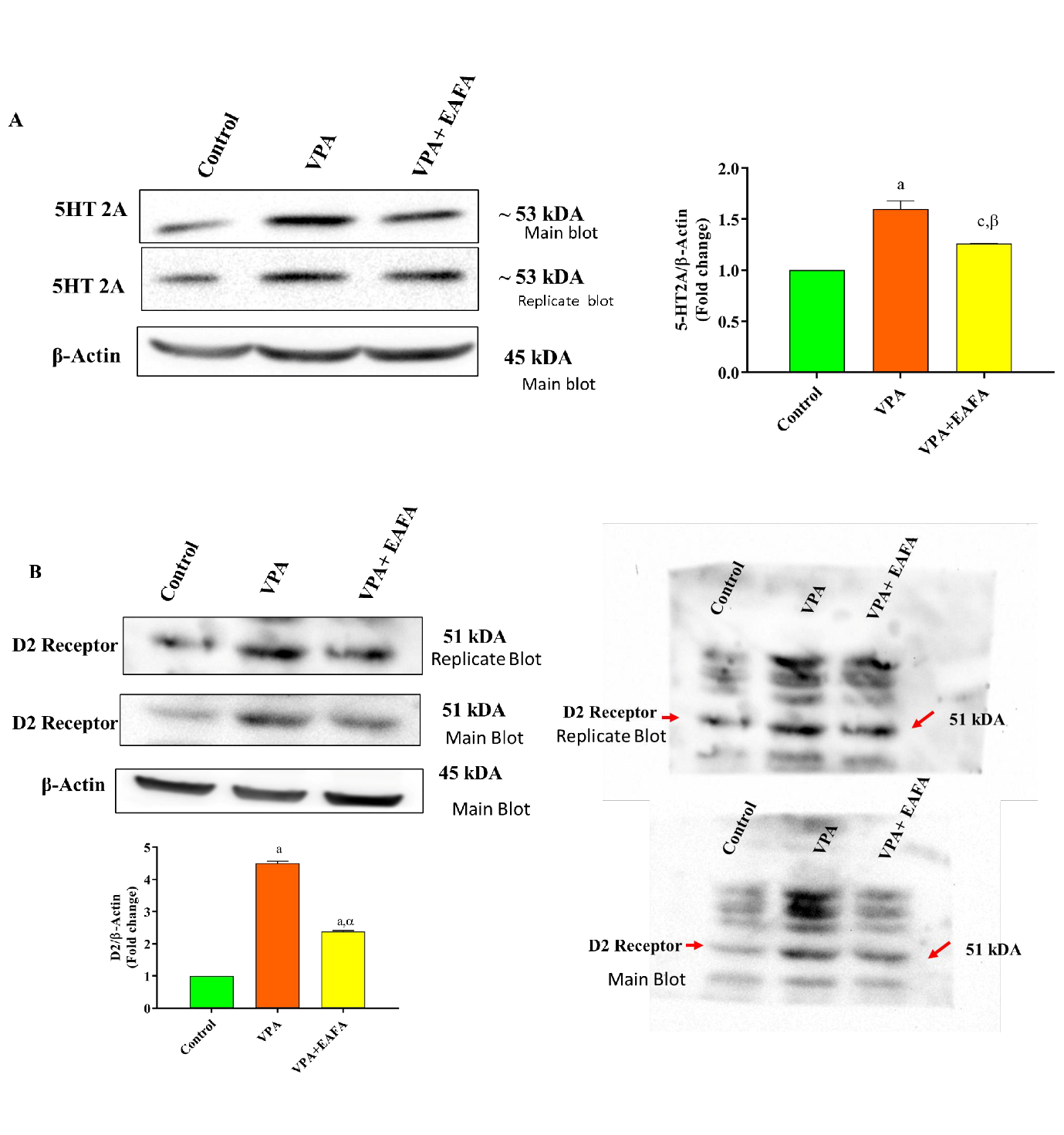
**

**Table S1.** The effect of reducing power assays at OD_700nm_ values on different *Phyllanthus emblica* extracts at different concentrations

| **Concentration mg/mL** | **EAFA (OD_700nm_)** | **70% methanol (OD_700nm_)** | **Vitamin C (OD_700nm_)** |
| --- | --- | --- | --- |
| 0.25 | 1.47 ±0.343 | 1.06 ± 0.008 | 1.27 ± 0.004 |
| 0.125 | 1.04 ± 0.013 | 0.60 ± 0.009 | 0.65 ± 0.030 |
| 0.0625 | 0.57 ± 0.017 | 0.31 ± 0.005 | 0.37 ± 0.006 |
| 0.03125 | 0.32 ± 0.012 | 0.16 ± 0.002 | 0.20 ± 0.002 |
| 0.015625 | 0.17 ± 0.015 | 0.10 ± 0.004 | 0.11 ± 0.004 |
| 0.007813 | 0.10 ± 0.008 | 0.07 ± 0.005 | 0.07 ± 0.001 |

**Table S2.** The list of compounds detected from the EAFA extract using UPLC-Q-TOF-MSE analysis.

|  | **Type of compound** | **Component name** | **Observed RT (min)** | **Expected mass (Da)** | **Observed mass (Da)** | **Observed m/z** | **Mass error (ppm)** | **Adducts** | **Molecular formula** |
| --- | --- | --- | --- | --- | --- | --- | --- | --- | --- |
| 1 | Amino acids | Cystine | 5.41 | 240.02 | 240.02 | 241.03 | 1.79 | +H, +K, +Na | C_6_H_12_N_2_O_4_S_2_ |
| 2 |  | arginine | 4.45 | 174.11 | 174.11 | 213.07 | -3.64 | +K | C_6_H_14_N_4_O_2_ |
| 3 |  | Glutamic acid | 4.42 | 147.05 | 147.05 | 206.07 | 2.55 | +CH3COO, +e, -H | C_5_H_9_NO_4_ |
| 4 | Vitamins | Vitamin A | 13.38 | 286.23 | 286.23 | 287.23 | -7.02 | +H | C_20_H_30_O |
| 5 |  | Vitamin C (Ascorbic acid) | 5.23 | 176.03 | 176.03 | 235.05 | 4.28 | +CH3COO | C_6_H_8_O_6_ |
| 6 |  | thiamin (Vitamin B1) | 18.28 | 265.11 | 265.11 | 266.12 | 5.43 | +H | C_12_H_17_N_4_OS+ |
| 7 |  | Folate (Vitamin B12) | 3.81 | 441.14 | 441.14 | 486.13 | -8.16 | +HCOO | C_19_H_19_N_7_O_6_ |
| 8 |  | Pantothenic Acid (Vitamin B5) | 6.21 | 219.11 | 219.11 | 242.10 | -2.83 | +Na | C_9_H_17_NO_5_ |
| 9 |  | Vitamin E | 22.97 | 430.38 | 430.38 | 469.34 | -2.27 | +K | C_29_H_50_O_2_ |
| 10 |  | Biotin (vitamin B7) | 4.29 | 244.09 | 244.09 | 283.05 | -4.88 | +K | C_10_H_16_N_2_O_3_S |
| 11 | Carbohyrate | d-fructose | 8.63 | 180.06 | 180.06 | 180.06 | 8.24 | -e | C_6_H_12_O_6_ |
| 12 |  | Pectin | 11.81 | 194.04 | 194.04 | 194.04 | -2.24 | -e | C_6_H_10_O_7_ |
| 13 | Tannins | Emblicanin A | 6.08 | 782.06 | 782.06 | 783.06 | -5.38 | +H | C_34_H_22_O_22_ |
| 14 |  | corilagin | 5.93 | 636.10 | 636.09 | 635.08 | -8.62 | -H, +HCOO, +Cl | C_27_H_24_O_18_ |
| 15 |  | Pedunculagin | 6.98 | 784.08 | 784.07 | 785.08 | -2.45 | +H | C_34_H_24_O_22_ |
| 16 |  | Geraniin | 6.10 | 952.08 | 952.08 | 970.11 | -2.87 | +NH4, +Na | C_41_H_28_O_27_ |
| 17 |  | Punigluconin | 5.41 | 802.09 | 802.08 | 803.09 | -2.72 | +H | C_34_H_26_O_23_ |
| 18 |  | Chebulagic acid | 5.45 | 954.10 | 954.09 | 953.08 | -7.83 | -H | C_41_H_30_O_27_ |
| 19 |  | Ellagotannin | 9.87 | 992.11 | 992.10 | 991.10 | -8.80 | -H | C_44_H_32_O_27_ |
| 20 |  | Chebulinic acid | 5.78 | 956.11 | 956.11 | 974.14 | -7.63 | +NH4, +Na | C_41_H_32_O_27_ |
| 21 |  | chebulic acid | 9.66 | 356.04 | 356.04 | 357.04 | -1.43 | +H, +Na | C_14_H_12_O_11_ |
| 22 |  | 1,2,3,4,6-penta-O-galloylβ-D-glucose | 7.85 | 940.12 | 940.12 | 939.11 | -1.88 | -H | C_41_H_32_O_26_ |
| 23 |  | 1,2,3,6-tetra-O-galloyl-β-D-glucose | 7.85 | 788.11 | 788.11 | 787.10 | 1.11 | -H | C_34_H_28_O_22_ |
| 24 |  | Emblicanin B | 15.09 | 780.04 | 780.05 | 781.05 | 1.68 | +H | C_34_H_20_O_22_ |
| 25 | Flavonoids | Quercetin | 9.98 | 302.04 | 302.04 | 303.05 | -3.74 | +H | C_15_H_10_O_7_ |
| 26 |  | Kaempferol | 8.02 | 286.05 | 286.05 | 287.05 | -2.32 | +H | C_15_H_10_O_6_ |
| 27 | Kaempferol derivatives | kaempferol 3-beta-d glucopyranoside | 8.03 | 448.10 | 448.10 | 449.11 | -0.20 | +H, +K, +Na | C_21_H_20_O_11_ |
| 28 |  | dihydrokaempferol | 11.03 | 288.06 | 288.06 | 323.03 | -5.69 | +Cl | C_15_H_12_O_6_ |
| 29 |  | kaempferol 3-o-rhamnoside | 9.76 | 432.11 | 432.10 | 433.11 | -2.28 | +H, +Na | C_21_H_20_O_10_ |
| 30 | Quercetin derivatives | quercetin 3-b-D-glucopyranoside | 5.41 | 463.09 | 463.09 | 481.12 | 4.35 | +NH4 | C_21_H_19_O_12_ |
| 31 |  | quercetin 3-O-glucoside | 3.83 | 464.10 | 464.10 | 463.09 | 2.91 | -H | C_21_H_20_O_12_ |
| 32 | Flavones | apigenin | 1.92 | 270.05 | 270.05 | 309.02 | 3.73 | +K | C_15_H_10_O_5_ |
| 33 |  | myricetin | 8.68 | 318.04 | 318.04 | 319.04 | -4.90 | +H | C_15_H_10_O_8_ |
| 34 | Flavanones | naringenin 7-O-glucoside | 8.03 | 434.12 | 434.12 | 435.13 | -2.24 | +H | C_21_H_22_O_10_ |
| 35 | Flavan-3-ols | gallocatechin | 11.67 | 306.07 | 306.07 | 365.09 | -5.38 | +CH3COO | C_15_H_14_O_7_ |
| 36 |  | epigallocatechin 3-O-gallate | 12.57 | 458.08 | 458.08 | 493.05 | -3.79 | +Cl | C_22_H_18_O_11_ |
| 37 | Polyphenols | Gallic acid | 7.76 | 170.02 | 170.02 | 171.03 | -9.69 | +H | C_7_H_6_O_5_ |
| 38 |  | Trigalloyl glucose | 6.17 | 636.10 | 636.09 | 635.08 | -9.24 | -H | C_27_H_24_O_18_ |
| 39 |  | ellagic acid | 8.67 | 302.01 | 302.01 | 303.01 | 1.89 | +H | C_14_H_6_O_8_ |
| 40 |  | Pyrogallol | 18.09 | 126.03 | 126.03 | 149.02 | 2.75 | +Na | C_6_H_6_O_3_ |
| 41 |  | Methyl gallate | 14.07 | 184.04 | 184.04 | 207.03 | 3.97 | +Na | C_8_H_8_O_5_ |
| 42 |  | Ethyl gallate | 14.07 | 198.05 | 198.05 | 221.04 | 2.26 | +Na | C_9_H_10_O_5_ |
| 43 | Other phenolic | Phenol, 3,5-bis (1,1-dimethylethyl)) | 22.94 | 452.33 | 452.34 | 470.37 | 3.47 | +NH4 | C_27_H_50_OP_2_ |
| 44 |  | mucic acid | 4.42 | 210.04 | 210.04 | 209.03 | 8.42 | -H | C_6_H_10_O_8_ |
| 45 | Gallic acid derivative | beta-glucogallin | 1.34 | 332.07 | 332.07 | 350.11 | -3.23 | +NH4, +H, +K, +Na | C_13_H_16_O_10_ |
| 46 | Turpinoids | β-carotene | 24.73 | 536.44 | 536.44 | 575.40 | 6.21 | +K | C_40_H_56_ |
| 47 | organic compounds | Stigmasterol | 20.58 | 412.37 | 412.37 | 413.37 | -7.60 | +H | C_29_H_48_O |

**Table S3.** The quantification of compounds in EAFA extract sample using LC-QqQ-MS.

| **S. No** | **Analyte** | **RT** | **Quantifier ion** | **CV/CE** | **R^2^** | **Concentration (µg/g) (Mean±SEM)** |
| --- | --- | --- | --- | --- | --- | --- |
| 1 | Ascorbic Acid | 4.25 | 177.06 > 14 | 30-Aug | 0.9928 | 69.29±1.81 |
| 2 | Quercetin | 6.26 | 303.1 > 153 | 45/32 | 0.999 | 7.88±0.078 |
| 3 | Gallic Acid | 3.16 | 169> 125 | 30-Dec | 0.9998 | 2.72±0.32 |
| 4 | Rutin | 5.11 | 611.2>303.1 | 38/15 | 0.9998 | 0.70±0.029 |
| 5 | Pantothenic acid | 4.77 | 220.1> 89.97 | 30-Dec | 0.9996 | 0.07±0.004 |
|  |  |  |  |  |  |  |

***CV = Cone voltage and CE = Collision energy RT= Retention time. *The values are based on triplicate analysis**
